# The AP-1 adaptor complex is required for cell surface modifications and the survival of *Cryptococcus neoformans* in phagocytic cells

**DOI:** 10.1128/iai.00098-26

**Published:** 2026-08-04

**Authors:** Eddy Sánchez-León, Kabir Bhalla, Victoria French, Polina Beskrovnaya, Shweta Parmar, Guanggan Hu, Melissa Lagace, Sophie Thanasack, E. Johan Foster, Elitza I. Tocheva, James W. Kronstad

**Affiliations:** 1 Michael Smith Laboratories, University of British Columbia 8166, Vancouver, BC, Canada; 2 Department of Microbiology and Immunology, University of British Columbia 8166, Vancouver, BC, Canada; 3 Department of Chemical and Biological Engineering, BioProducts Institute, University of British Columbia 8166, Vancouver, BC, Canada; 4 Life Sciences Institute, University of British Columbia 8166, Vancouver, BC, Canada; University of Illinois Chicago, Chicago, Illinois, USA

**Keywords:** endomembrane trafficking, phagocytosis, virulence, fungal pathogenesis

## Abstract

The pathogenic yeast *Cryptococcus neoformans* causes life-threatening meningoencephalitis in individuals with compromised immune systems. The ability of the fungus to cause disease depends on key cell-surface features, such as a polysaccharide capsule that protects it from the mammalian immune system. However, the mechanisms by which *C. neoformans* traffics polysaccharide capsule, melanin, and other materials to the cell surface are poorly understood. In this study, we employed mutants lacking specific subunits of the adaptor protein complex 1 (AP-1) to investigate its role in the elaboration of virulence-related materials at the cell surface. Importantly, the mutants displayed multiple defects, including alterations in capsule size and cell morphology, and defects in melanin production and urease secretion. Together, these results support the key observation that the AP-1 complex is required for *C. neoformans* survival in phagocytic cells. Together, our findings provide insights into the endomembrane trafficking machinery required for fungal pathogenesis.

## INTRODUCTION

*Cryptococcus neoformans*, the causative agent of cryptococcal meningoencephalitis, is ranked as a “critical” fungal pathogen on the World Health Organization’s (WHO) Fungal Priority Pathogens List (FPPL) (1, 2). The fungus primarily affects individuals with compromised immune systems, with people living with HIV/AIDS being among the most affected (3–6). Additionally, *C. neoformans* poses a threat to non-HIV individuals with underlying conditions, including diabetes mellitus, renal failure, viral respiratory tract infections, such as SARS-CoV-2, and other chronic diseases (6–8). Recent estimates indicate that the annual global incidence of cryptococcal meningitis is approximately 194,000 cases, resulting in ~118,000 deaths (2). Due to high antifungal drug resistance and mortality rates ranging from 41% to 61%, there is a growing need for a detailed understanding of virulence traits and disease mechanisms to improve therapeutic approaches (9). *C. neoformans* has a unique arsenal of complex virulence traits such as the polysaccharide capsule and melanin production, and our current study focuses on trafficking functions required for elaboration of these cell surface factors (10–14).

Key trafficking functions in eukaryotic cells include five adaptor protein complexes (AP-1 to AP-5) that mediate transport between distinct donor and target membrane compartments (15, 16). These heterotetrameric adaptor protein complexes are distributed across specific subcellular compartments and function in regulating vesicular trafficking. By recognizing sorting motifs on cargo proteins, they mediate their packaging into vesicular carriers, which often recruit clathrin and accessory proteins to drive downstream transport processes (15, 17). Composed of four distinct subunits, including two large subunits known as adaptins (and termed β-adaptin and γ-, α-, δ-, ε-, or ζ-adaptin), one medium-sized μ subunit, and one small σ subunit, the AP complexes have crucial roles in the endocytic and secretory pathways (10, 15, 18–20).

In addition to its established role in mediating endocytic trafficking and cargo recycling to the Golgi, the AP-1 complex has also been implicated in regulating protein polarization across diverse organisms (20–24). For instance, AP-1–mediated plasma membrane protein transport has been reported in mammalian epithelial cells and in *Caenorhabditis elegans* (21, 25). However, polarized secretion does not appear to be restricted to metazoans, as studies in *Arabidopsis thaliana* have shown that this adaptor complex participates in the polar sorting of mucilage in seed coat cells (23). In fungi, the AP-1 complex has been linked to the polarized trafficking of secretory vesicles toward hyphal apices (22). Although the diverse roles of the AP-1 complex have been extensively characterized across various eukaryotic systems, its contribution to the development of virulence factors in fungal pathogens remains poorly understood. Recent studies in pathogenic fungi have described the trafficking functions of this adaptor complex, underscoring its roles in virulence factor secretion, cell wall integrity, and protein polarization (26–29). In addition, inactivation of the AP-1 complex in malaria parasites causes a growth defect, and the complex also plays a role in protein sorting (30, 31).

In previous work, we identified a role for ER-to-Golgi protein transport and the trans-Golgi and post-Golgi compartments in the elaboration of the polysaccharide capsule, the major virulence factor of *C. neoformans* (10). Specifically, the inhibitors brefeldin A and monensin blocked capsule formation, thus confirming that AP-1 facilitates bidirectional trafficking between organelles and contributes to the recycling of cargo from endosomes to the plasma membrane (10). To examine the mechanisms underlying the role of the endomembrane trafficking machinery in capsule formation, we targeted AP-1, which is localized to the *trans*-Golgi network (TGN) and tubular early endosomes (15, 17, 22, 32). Specifically, we characterized mutants deficient in the large (*apl2*∆ and *apl4*∆), medium (*apm1*∆), and small (*aps1*∆) subunits of the AP-1 complex. With these mutants, we discovered that each subunit of the AP-1 complex is required for survival of the fungus in phagocytic cells. We propose that the defect in survival is due to impaired trafficking of cell surface and vacuole-associated proteins to influence virulence traits such as the polysaccharide capsule, melanin, and secreted enzymes such as urease and acid phosphatase. A recent study also revealed that the subunits of the AP-1 complex make distinct contributions to the virulence of the fungus in a mouse model of cryptococcosis (33). Moreover, the loss of individual AP-1 components induced distinct pleiotropic phenotypes under stress conditions, highlighting the multifaceted role of this complex in *C. neoformans*.

## RESULTS

### Disruption of the AP-1 complex alters virulence factor expression

**Fig 1:**
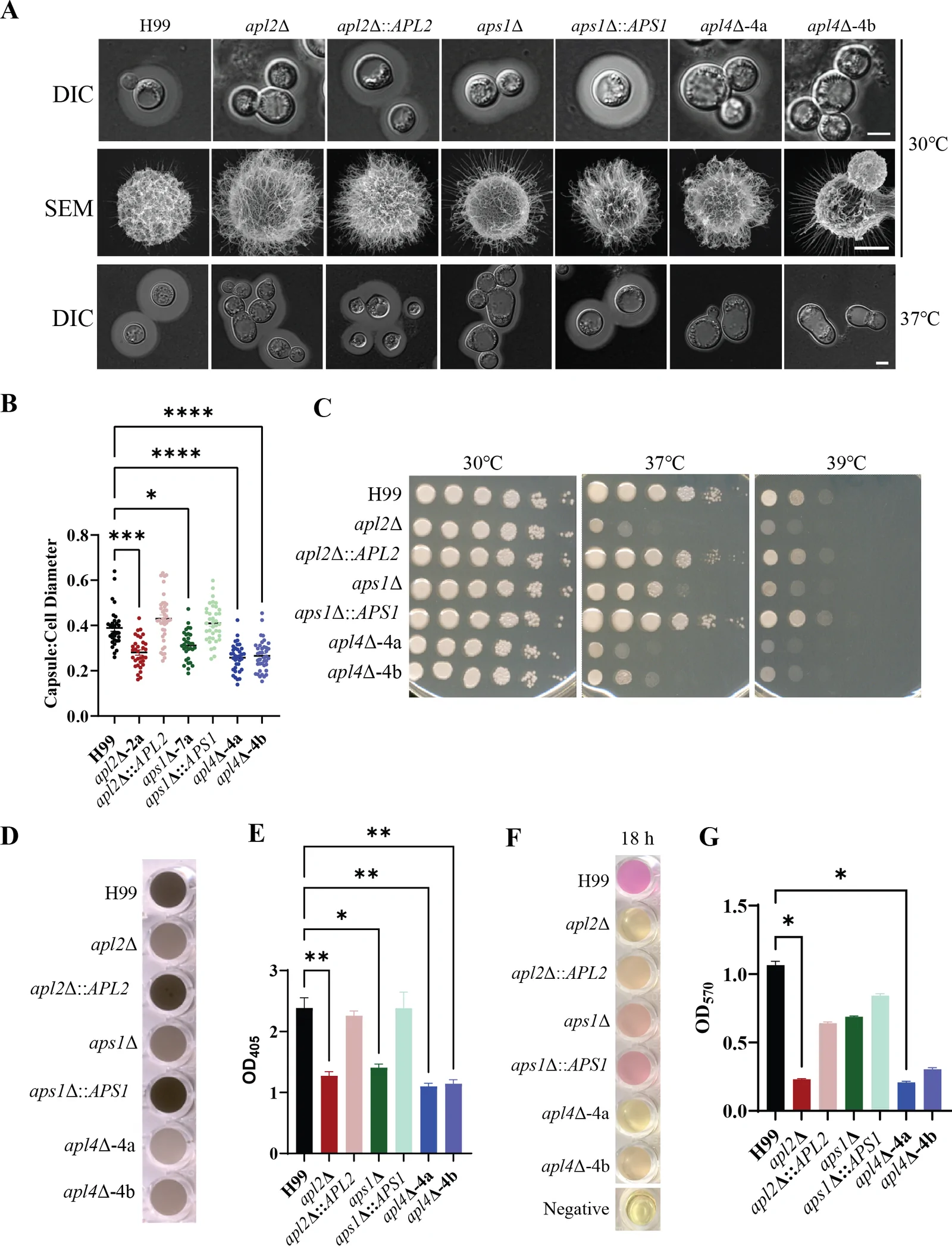
Loss of AP-1 subunits affects cellular morphology and virulence factor development. (**A**) Differential interference contrast (DIC) microscopy of wild-type (H99), single deletion mutants (*apl2*Δ, *aps1*Δ, *apl4*Δ-4a, and *apl4*Δ-4b), and complemented strains (*apl2*Δ::*APL2* and *aps1*Δ::*APS1*). Cells (1 × 10³ cells/mL) were cultured in defined low-iron medium (dLIM) for 48 h at 30°C or 37°C and stained with India ink to visualize the polysaccharide capsule. SEM images were obtained under identical capsule-inducing conditions. Scale bars (5 µm). (**B**) Capsule-to-cell diameter ratios of the corresponding strains were obtained from cells grown in capsule-inducing conditions, and data represent individual values (*n* = 100) and the average of three independent experiments ± standard deviation (SD). Statistical significance was determined by ANOVA followed by Kruskal-Wallis *post hoc* test (*, *P* < 0.05; ***, *P* < 0.001; ****, *P* < 0.0001). (**C**) Tenfold serial dilutions of the indicated strains were spotted onto solid YPD medium and incubated at 30°C, 37°C, and 39°C for 2–3 days. Plates were then scanned, and representative images from at least three independent experiments are shown. (**D and E**) Melanin production assessed in cells cultured overnight in defined L-DOPA liquid medium. Quantification of melanin in the culture supernatants was performed at 405 nm spectrophotometrically after normalization. (**F and G**) Urease secretion assays were performed using the indicated strains. Cells (2 × 10³ cells/mL) were cultured in Christensen’s urea broth for 18 h. Following normalization of cell densities, urease activity in the culture was quantified spectrophotometrically at 570 nm. Data in (**E**) and (**G**) represent the averages of three independent experiments ± SD. Statistical significance was determined by ANOVA followed by Kruskal-Wallis *post hoc* test (*, *P* < 0.05; **, *P* < 0.01).

Considering the crucial influence of the AP-1 complex on protein trafficking (20, 31), we hypothesized that deletion of genes encoding AP-1 components would impact the cell surface and elaboration of major virulence factors in *C. neoformans*. To investigate this possibility and further examine the role of the AP-1 complex, we generated deletion mutants lacking each of the two large subunits (*apl2*Δ and *apl4*Δ) or the small subunit (*aps1*Δ) of the complex. Complemented strains were also constructed for the *aps1*Δ and *apl2*Δ mutants, and two independent mutants were examined for the *apl4*Δ deletion. Mutants lacking the medium subunit are discussed below. We found that the mutants lacking the small or large subunits had significant alterations in capsule size and morphology compared to the wild-type strain H99 (WT) (Fig. 1). Specifically, the mutants displayed reduced capsule-cell diameter ratios compared to WT (strain H99) and the complemented strains *apl2*Δ::*APL2* or *aps1*Δ::*APS1* upon growth in capsule inducing medium (CIM) (Fig. 1A and B; Fig. S1). To determine whether these phenotypes were conserved under host-like conditions, we analyzed capsule-to-cell diameter ratios in cells grown in other media (RPMI or DMEM). Under these host-mimicking conditions, the mutants and complemented strains exhibited a similar defect in capsule production (Fig. S2), consistent with the phenotype observed in CIM (Fig. 1A; Fig. S1). Scanning electron microscopy (SEM) further revealed that capsule architectures were altered for the deletion mutants grown at 30°C. For example, WT cells exhibited a compact and continuous capsule surface, with fibrillar projections extending radially from the cell body toward the capsule periphery (Fig. 1A). In contrast, the deletion mutants displayed capsules with a less compact surface organization and fibrillar structures that appeared irregular in morphology and length. These observations implicate the AP-1 complex in polysaccharide capsule development in *C. neoformans*. Together with evidence from *A. thaliana* demonstrating a role for the AP-1 complex in directing mucilage transport to the outer surface of seed coat cells, our results support a conserved function for the complex in the production and/or delivery of extracellular material (23).

**Fig 2:**
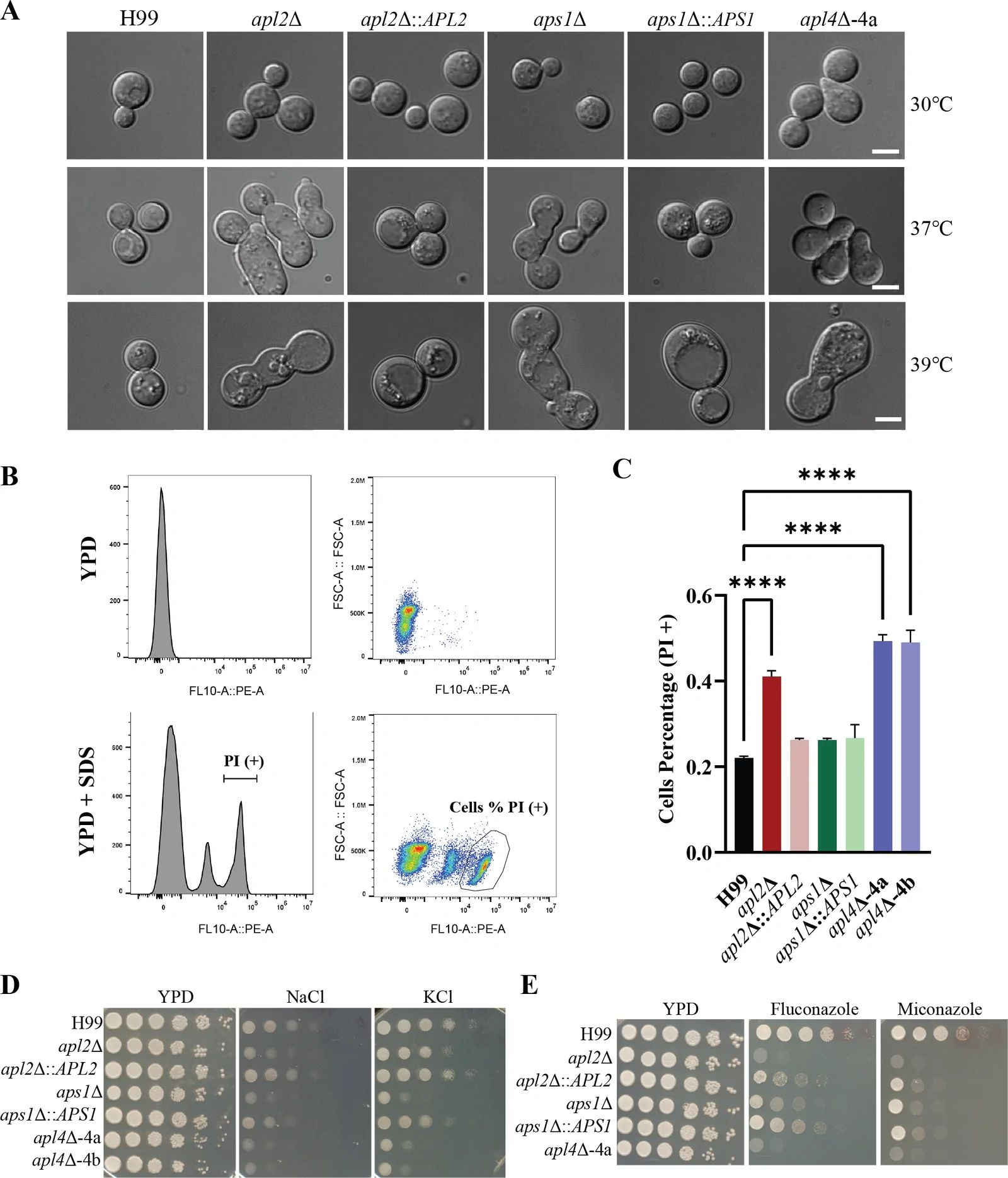
Disruption of the AP-1 complex alters cellular morphology and increases sensitivity to membrane stress. (**A**) DIC microscopy of wild-type (H99), single deletion mutants (*apl2*Δ, *aps1*Δ, *apl4*Δ-4a, and *apl4*Δ-4b), and complemented strains (*apl2*Δ::*APL2* and *aps1*Δ::*APS1*). Cells were cultured in YPD medium overnight at 30°C, 37°C, and 39°C. Images are representative of at least three independent experiments, each observing more than 100 cells. Scale bars (5 µm). (**B and C**) Flow cytometry analysis of membrane permeability in the indicated strains. Cells were incubated in YPD medium with or without 0.05% sodium dodecyl sulfate (SDS) for 3 h and stained with propidium iodide (PI, 2.5 μg/mL). Data represent the mean percentage of PI-positive cells ± standard error of the mean (SEM) from three independent experiments. The proportion of PI-positive cells was quantified using the gating strategy illustrated in Fig. S2B. Statistical significance was determined by one-way ANOVA followed by Tukey’s *post hoc* test (****P < 0.0001). (**D and E**) Tenfold serial dilutions of the indicated strains were spotted onto solid YPD medium supplemented with high salt concentrations (1.5 mM NaCl or 1.5 mM KCl) or the azole antifungals fluconazole (10 µg/mL) and miconazole (0.01 µg/mL). Plates were incubated at 30°C for 2–3 days before being scanned. Representative images from at least three independent experiments are shown.

Thermotolerance is a critical virulence trait in *C. neoformans* as it directly influences growth and persistence at host physiological temperature (≥37°C) (34–36). Serial dilution assays showed that AP-1 deletion mutants (*apl2*Δ, *apl4*Δ, and *aps1*Δ) presented marked growth defects at 37°C and 39°C compared to WT or complemented cells (Fig. 1C). These defects were more pronounced in mutants lacking the large subunits of the complex compared with the *aps1*Δ mutant and were further exacerbated at elevated temperatures for the *apl2*Δ and *apl4*∆ mutants where capsule size was most strongly affected (Fig. 1A). Similar patterns of growth were observed for the mutants in RPMI or DMEM (Fig. S3).

In *C. neoformans,* the deposition of melanin in the cell wall confers protection against different environmental stressors and, importantly, contributes to the ability of the fungus to survive and proliferate within the host, thereby influencing disease (37, 38). Melanin measurements indicated that loss of any subunit of the complex reduced production by 50% or more (Fig. 1D and E). *C. neoformans* also secretes urease, an enzyme whose activity facilitates dissemination to the host brain (39, 40). In particular, the fungus secretes high levels of urease during infection, and the enzyme hydrolyzes urea into ammonia and carbon dioxide, leading to multiple host alterations. These include yeast sequestration in the microvasculature, modification of phagolysosome pH, tissue damage, and hyperammonemia (41–43). Since AP-1 disruption impacts the secretion-dependent processes of capsule and melanin elaboration (44–46), we tested whether loss of AP-1 subunits also compromises urease secretion. Our assays revealed a marked reduction in extracellular urease secretion and activity in all the AP-1 deletion mutants compared to WT or complemented cells, with the strongest defects observed in *apl2*Δ and *apl4*Δ mutants (Fig. 1F and G). These findings indicate that the AP-1 complex contributes to the efficient secretion of urease, further underscoring its central role in regulating secretory-dependent virulence factors in *C. neoformans*.

To complete our analysis of the AP-1 complex, we also investigated the role of the medium subunit. We generated two independent deletion strains, *apm1*∆-3a and *apm1*∆-6b, and examined capsule formation, thermotolerance, and melanin production to assess whether loss of the medium subunit reproduces the phenotypes observed in mutants lacking the large and small subunits (Fig. S4). The *apm*1Δ strains exhibited defects similar to those of the mutants lacking the large subunits, further emphasizing the importance of Apm1 in AP-1 complex function. Taken together, these results suggest that the AP-1 complex in *C. neoformans* plays a critical role in the elaboration of major virulence factors at the cell surface.

### AP-1 mutants exhibit altered morphology and increased sensitivity to cell wall and membrane stress

Given the observed defects in polysaccharide capsule elaboration, melanin production, and urease secretion, we hypothesized that the AP-1 complex might also contribute to cell morphogenesis, likely through defective transport of membrane components and cell wall materials. Consistent with this prediction, each of the AP-1 subunit mutants displayed enlarged cells with large central vacuoles and cell separation defects resulting in cells with multiple buds (Fig. 2A; Fig. S2A and S5A). Loss of the WT morphology was further exacerbated under stress conditions, such as elevated temperature, with the frequent appearance of multiple budded cells and pseudo-hyphae-like structures.

We next examined whether the observed morphological changes were associated with compromised plasma membrane integrity. In *Saccharomyces cerevisiae*, loss of Mid2p, a cell wall integrity (CWI) pathway sensor, affects plasma membrane and cell wall integrity, influencing morphology and cell viability during polarization (47). We assessed the impact of loss of AP-1 complex subunits on plasma membrane integrity by exposing the mutants to SDS and osmotic stress. Flow cytometry and serial dilution growth assays revealed that all AP-1 deletion mutants were more sensitive to SDS than the WT or complemented strains, indicating a role in plasma membrane integrity (Fig. 2B and C; Fig. S5B and C). The mutants were also sensitive to 1.5 M NaCl and 1.5 M KCl, thus indicating involvement in osmotic stress tolerance (Fig. 2D). Given the observed phenotypes related to plasma membrane integrity, we next examined whether the complex also influences the response to azoles. Azole antifungal drugs target the ergosterol biosynthetic pathway, leading to alteration in membrane composition and accumulation of toxic sterols (48–50). We observed that both fluconazole and miconazole significantly reduced the growth of the *apl2*Δ, *apl4*∆, and *aps1*Δ mutants (Fig. 2E). We confirmed that the same defect was present in *S. cerevisiae* cells deficient in AP-1 by performing similar tests on *apl2*Δ and *aps1*Δ mutants. These mutants exhibited increased sensitivity to azoles, although the effect was less pronounced than for the *C. neoformans* mutants (Fig. S5D). The enhanced sensitivity to azoles of AP-1 mutants suggests changes in either the membrane stability/composition of the cells or potentially altered positioning of efflux transporters at the cell surface.

The cell morphology defects of the mutants could also be linked to abnormalities in the cell wall. A role of the AP-1 complex in the regulation and integrity of the cell wall has previously been reported in other fungi (22, 26–28). We therefore investigated whether defects in the complex caused increased sensitivity to the cell wall inhibitors caffeine and calcofluor white (Fig. S6A). Caffeine activates the CWI signaling pathway in yeast cells, and sensitivity to this drug is associated with cell wall defects (51, 52). We found that growth inhibition was moderately affected only in the mutants lacking the large subunits. To further investigate the effect of cell wall inhibitors on AP-1 localization, we analyzed the cellular distribution of Apl2 tagged with the red fluorescent protein mKATE2. The functionality of the Apl2 fusion protein was confirmed by complementation of WT phenotypes in the *apl2*Δ mutant (Fig. S6B). In contrast to untreated cells, caffeine treatment caused a diffuse cytoplasmic distribution of Apl2-HA-mKATE2 with markedly fewer punctate structures than in control samples (Fig. S6C). Interestingly, exposure to calcofluor white (CFW) enhanced punctate formation, indicating a possible role of cell wall stress in modulating AP-1 localization. Taken together, these findings underscore the importance of the AP-1 complex in processes linked to the plasma membrane and cell wall integrity.

### Loss of AP-1 function impairs transport of acid phosphatase and a capsule-associated protein

**Fig 3:**
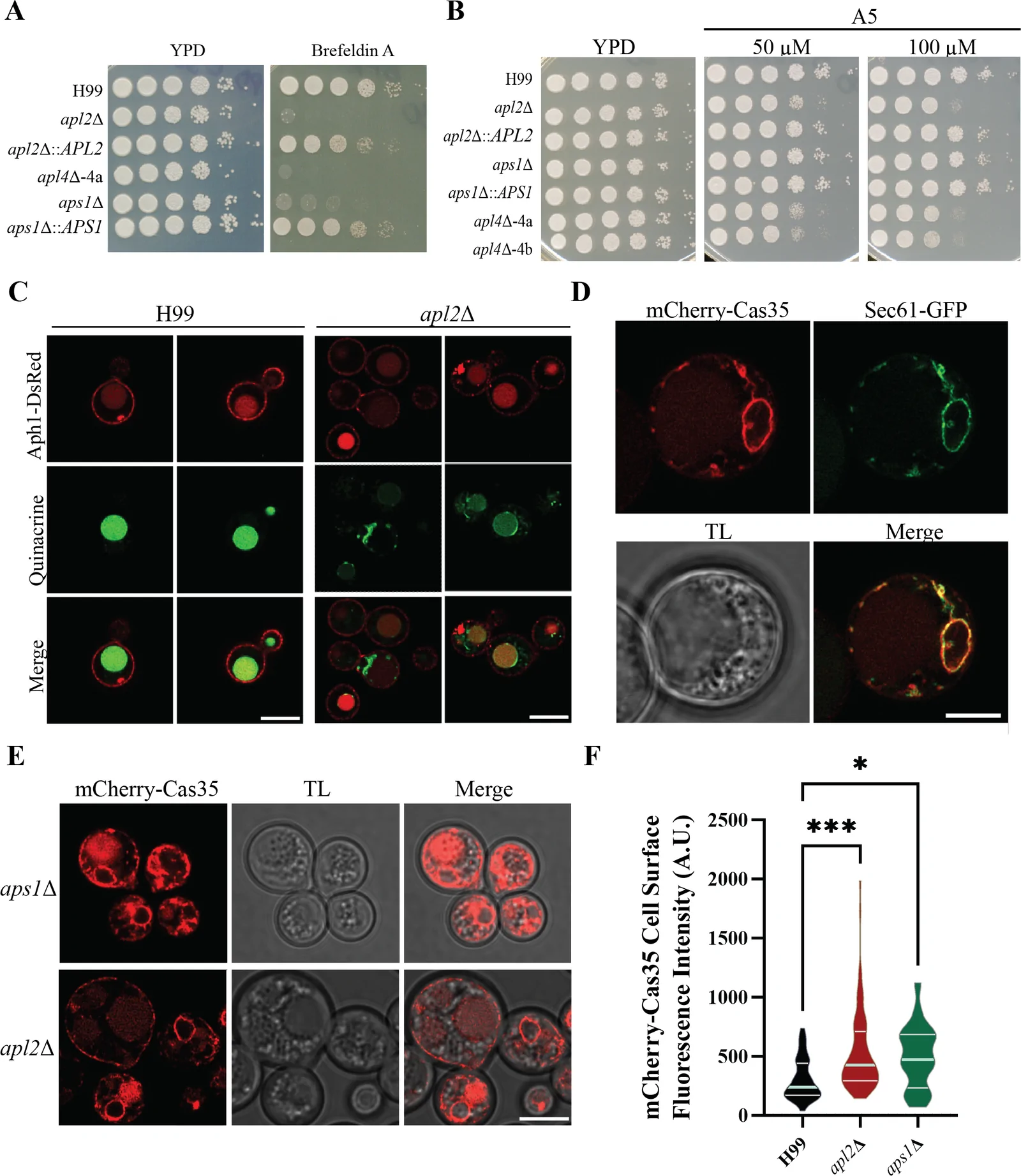
Impaired AP-1 complex function leads to defective secretion of cell surface proteins. (**A and B**) Tenfold serial dilutions of the indicated strains were spotted onto solid YPD medium supplemented with or without the secretory inhibitors brefeldin A (BFA, 30 μg/mL) or A5. Serial dilution plates were incubated at 30°C for 2–4 days before being scanned. Representative images from at least three independent experiments are shown. Note that the control YPD plate without any additions was also part of the experiment shown in Fig. 4B. (**C**) Laser scanning confocal microscopy of wild-type and *apl2*Δ strains expressing Aph1-DsRed. Cells were stained with quinacrine dihydrochloride (200 μM) for 10 min in H3G buffer before imaging. Scale bars, 5 μm. (**D and E**) Laser scanning confocal microscopy of wild-type and AP-1 deletion strains (*apl2*Δ and *aps*1Δ) expressing Sec61-GFP with or without the Cas35 fused with mCherry. Scale bars, 5 μm. Transmitted light (TL). (**F**) Quantification of Cas35-mCherry fluorescence of regions of interest (0.950 μm^2^) at the cell surface. Violin plots show fluorescence intensity (A.U.) of Cas35-mCherry at the cell periphery in wild-type (H99) and AP-1 mutant strains (*apl2*Δ and *aps1*Δ). Bars indicate median and interquartile ranges. Statistical significance was determined by one-way ANOVA followed by Tukey’s *post hoc* test (*P < 0.05; ***P < 0.001).

The AP-1 complex has been reported to mediate polarized secretion and membrane trafficking between the Golgi and endosome (22, 23, 26, 27, 53–56). Brefeldin A (BFA) is a fungal metabolite that indirectly inhibits AP-1 complex activity by targeting the activator ADP-ribosylation factor Arf1 (57, 58). As mentioned, we previously found that BFA inhibits capsule formation, and additional studies identified sensitivity in *C. neoformans* mutants defective in capsule and/or melanin production (10, 59). By disrupting membrane trafficking at a critical step of the secretory pathway, BFA interferes with cargo transport to and from the plasma membrane (10, 60, 61). Given the defective activity for secreted urease in the AP-1 complex mutants (Fig. 1F and G), we investigated whether the complex influences other secretory activities. We first found that each of the deletion mutants was highly sensitive to BFA in serial dilution growth assays, displaying dramatically reduced growth compared to the WT strain (Fig. 3A). To further confirm whether the observed BFA trafficking defects were associated with AP-1 activity, we next assessed the growth of the mutants in the presence of the compound A5 (Fig. 3B). Studies on mammalian cells demonstrate that A5, a piperazinyl phenylethanone-based compound, selectively disrupts the trafficking activities regulated by the AP-1 complex (62). Mutants lacking the large subunits of the complex displayed increased sensitivity to A5 compared to the WT and complemented strains. Interestingly, the *aps1*Δ mutant exhibited growth comparable to that of the WT and complemented strains.

The impaired secretory function in the mutants was further confirmed by examining the cellular localization of acid phosphatase 1 (Aph1). Previous studies have shown that Aph1 localizes to the cell periphery and vacuoles, suggesting roles in extracellular phosphate scavenging and vacuolar phosphate recycling (63). Fluorescence microscopy of Aph1-DsRed in the AP-1 defective strain (Aph1-DsRed::*apl2*Δ) revealed a marked reduction of the protein at the cell surface in contrast to the WT strain. Interestingly, the overall presence of Aph1-DsRed at the vacuoles was not altered (Fig. 3C). The defects in capsular architecture and size observed in the mutants suggested that capsular-associated proteins or materials may fail to reach the cell surface due to trafficking defects (Fig. 1). We tested this idea by examining the distribution of the capsule-associated protein Cas35. Several studies indicate that loss of Cas35 in *C. neoformans* negatively impacts capsule production and virulence (64–66). We examined Cas35 localization by fusing the protein to the mCherry fluorescent protein and expressing the fusion in a WT strain carrying Sec61-GFP, as well as in AP-1 deletion mutants (*aps1*Δ and *apl2*Δ). Sec61 protein is an essential subunit of the Sec61 translocon complex channel in the endoplasmic reticulum, which mediates the translocation and membrane integration of proteins destined for the secretory pathway (67). We performed super-resolution confocal microscopy to examine the subcellular localization of the fusion protein (Fig. 3D). Interestingly, in WT cells, mCherry-Cas35 localized to perinuclear regions and accumulated in discrete puncta at the cell periphery, where it colocalized with Sec61-GFP. Like WT cells, mCherry-Cas35 remained around the nuclei in the AP-1 defective strains; however, the mutants exhibited increased cell surface accumulation, which appeared either at the cortical ER or at the plasma membrane (Fig. 3E). Consistent with these observations, quantification of fluorescence at the cell surface revealed significantly higher intensity in the *apl2*Δ and *aps1*Δ cells compared to WT (Fig. 3F). Taken together, these results suggest that the loss of AP-1 impairs trafficking of proteins to the cell surface. The secretory defects evidenced by hypersensitivity to BFA and A5, the mislocalization of Aph1-DsRed, and the altered distribution of Cas35 further suggest that AP-1 regulates the delivery of capsule-associated and extracellular secreted proteins.

### Defective AP-1 complex subunits lead to impaired vacuolar activity

**Fig 4:**
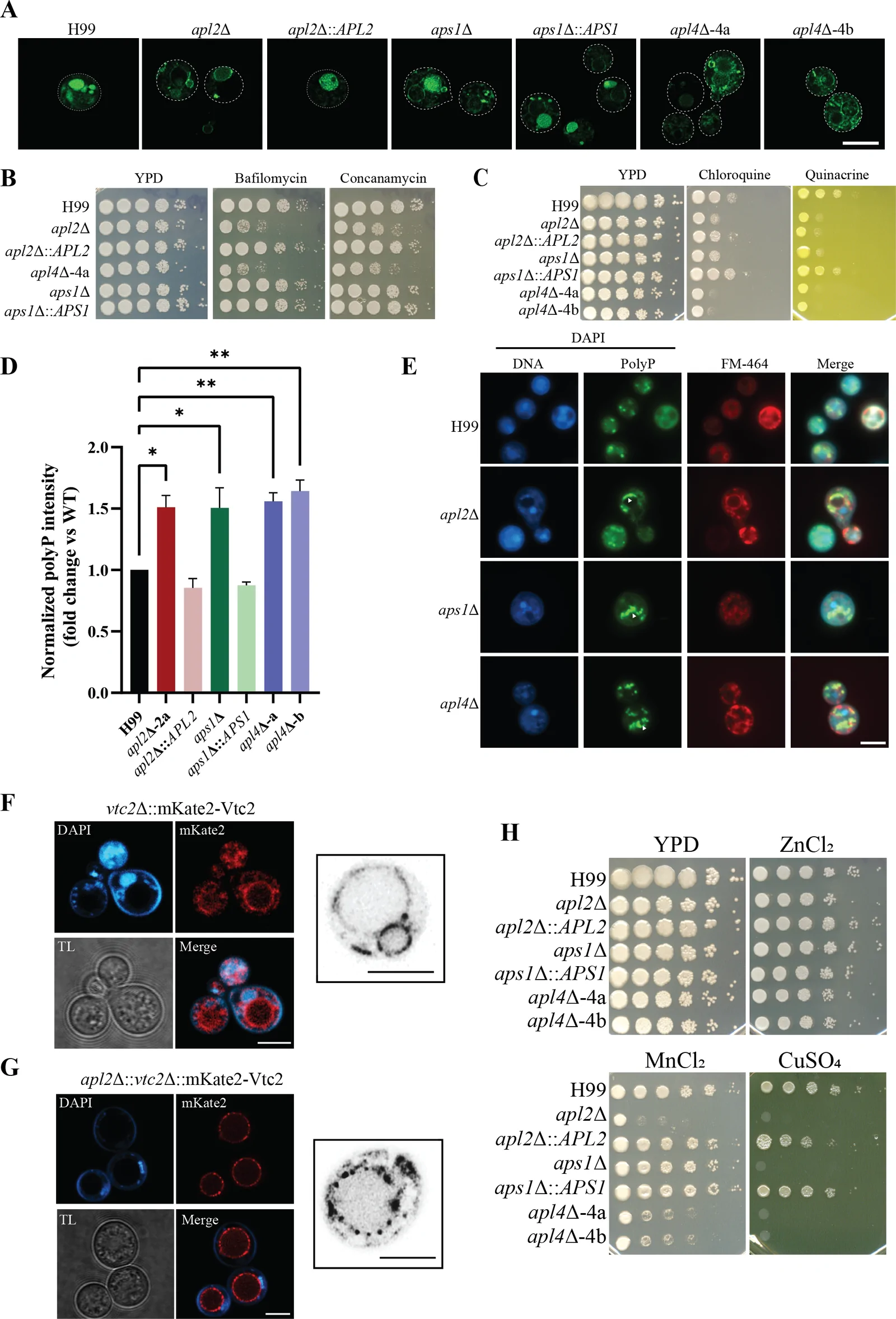
Hyperaccumulation of polyP and dysregulation of vacuolar functions are related to AP-1 complex defects. (**A**) Laser scanning confocal microscopy of wild-type (H99), single deletion mutants (*apl2*Δ, *aps1*Δ, *apl4*Δ-4a, and *apl4*Δ-4b), and complemented strains (*apl2*Δ::*APL2* and *aps1*Δ::*APS1*) stained with quinacrine dihydrochloride (200 μM) for 10 min in H3G buffer as in Fig. 3C. Scale bars, 5 μm. (**B and C**) Tenfold serial dilutions of the indicated strains spotted onto solid YPD medium supplemented with or without the vacuole function inhibitors bafilomycin A (1 μM), concanamycin (500 nM), chloroquine (6 mM), or quinacrine (1.6 mM). Growth assay plates were incubated at 30°C for 2–4 days before being scanned. Representative images from at least three independent experiments are shown. Note that the control YPD plate without any additions was also part of the experiment shown in Fig. 3A. (**D**) Densitometric analysis from acrylamide gels stained for polyP. Measurements correspond to data obtained from lane regions containing polyP from the indicated strains normalized to the values obtained from wild type (H99). The results represent the mean from three independent experiments ± SEM. Statistical significance shown was determined by ANOVA followed by Tukey *post hoc* test (*, *P* < 0.05; **, *P* < 0.01). (**E**) Wide-field fluorescence microscopy of the indicated strains showing cells stained with FM4-64 (5–10 μm) and DAPI (100 μg/mL) for 30 min at room temperature before imaging. Images were captured with filter sets (Ex/Em 572/645 nm) for FM4-64-stained membranes, DAPI (Ex/Em 359/461 nm) filter sets for DNA, and the BrightLine (Ex/Em 407/530 nm) full multiband filter set for polyP. Scale bar, 5 μm. (**F and G**) Laser scanning confocal microscopy of AP-1 deletion strain *apl2*Δ expressing mKate2-Vtc2 in a *vtc2*Δ background. Cells were stained with DAPI (2–5 μg/mL) for 10–30 min at room temperature before imaging. Inverted side images (right panels) are shown to enhance visualization of mKate2-Vtc2 localization. Merged panels include transmitted light images (not shown). Scale bars, 5 μm. (**H**) Tenfold serial dilutions of the indicated strains spotted onto solid YPD medium supplemented with or without zinc chloride (ZnCl_2_, 2.5 mM), copper sulfate (CuSO_4_, 10 mM), or manganese chloride (MnCl_2_ · 4H_2_O, 3.75 mM). Plates were incubated at 30°C for 2–4 days before being scanned. Representative images from at least three independent experiments are shown.

The role of the AP-1 complex in trafficking vacuolar and lysosomal proteins has been reported in several studies (68–71). Although its specific involvement in post-Golgi to endosome transport pathways and trafficking to the plasma membrane remains debated (17), disruptions in protein sorting to the vacuole, whether direct or indirect, are a consistent feature of AP-1-deficient mutants. For example, a study with *S. cerevisiae* deletion mutants demonstrated that the AP-1 complex is required for the transport of ubiquitylated cargoes to vacuoles (69). We therefore examined vacuolar functions in our *apl2*Δ, *apl4*Δ, and *aps1*Δ deletion strains. Quinacrine and chloroquine are weakly basic amines that accumulate in acidic compartments such as vacuoles, and both are used to determine alterations in organelle physiology. Microscopy analysis revealed that vacuolar acidification was specifically impaired in the deletion mutants lacking the large subunits of the complex, as evidenced by the absence of quinacrine accumulation within vacuoles in *apl2*Δ and *apl4*Δ strains (Fig. 4A). Conversely, *aps1*Δ cells accumulated quinacrine and exhibited a vacuolar phenotype similar to that of the WT strain. Given that acidification of the vacuolar lumen was altered in AP-1 deletion mutants, we further investigated whether organelle functions were also disrupted. Serial dilution assays on media containing the vacuolar function inhibitors bafilomycin A or concanamycin revealed significant growth defects in mutants lacking the medium or large subunits of the complex, consistent with the microscopy observations (Fig. 4A and B). Interestingly, perturbation of vacuolar physiology upon exposure to chloroquine or quinacrine impaired growth in all of the AP-1 mutants, including *aps1*Δ and *apm1*Δ, indicating that the small and medium subunits also contribute to AP-1 complex functionality at the vacuolar level (Fig. 4C; Fig. S7A).

### AP-1 complex defects cause polyphosphate accumulation and divalent cation sensitivity

Previously, we demonstrated that the HOPS complex in *C. neoformans,* which is involved in late endocytic steps, has roles in endomembrane trafficking to the vacuole, including the vCLAMPs contact sites between vacuoles and mitochondria (12, 72). That is, we found that loss of the HOPS subunit Vam6 led to pleiotropic phenotypes, including dysregulation of polyphosphate (polyP) metabolism and mitochondrial function. The importance of polyP homeostasis in *C. neoformans* was further underscored by our recent report that mutants hyperaccumulating polyP exhibited virulence defects (73). Given that the AP-1 complex influences vacuolar functions and that polyP regulation is linked to late endomembrane trafficking, we hypothesized that defects in AP-1 may lead to alterations in polyP accumulation and its subcellular distribution. To assess these changes, RNA and polyP from whole-cell lysates were visualized on polyacrylamide gels stained with toluidine blue O and quantified by densitometric analysis (Fig. 4D; Fig. S7B and C). We found that each of the mutants displayed elevated polyP levels compared to WT. Specifically, *apl2*Δ, *apl4*Δ, and *aps1*Δ accumulated ~50% more polyP, as quantified by densitometric analysis. Fluorescence microscopy of DAPI-stained cells further revealed aberrant accumulation of polyP granules in the mutants (Fig. 4E). These granules were primarily localized within membrane-bound compartments visualized by FM4-64 staining. Interestingly, DAPI-stained polyP structures were also observed to accumulate in proximity to the vacuolar membrane. PolyP biosynthesis in *C. neoformans* requires the activity of the VTC complex, which consists of at least three subunits (Vtc1, Vtc4, and either Vtc2 or Vtc3), and our recent study revealed that the functional fusion protein mKate2-Vtc2 localizes to polyP granules, vacuoles, perinuclear regions, and the cell periphery (72–75). To investigate whether disruption of AP-1 affects Vtc2 localization, we performed confocal fluorescence microscopy on the *apl2*Δ mutant expressing the mKate2-Vtc2 fusion protein. Consistent with the identified alterations of cellular content of polyP in the mutants, loss of Apl2 altered the subcellular distribution of mKate2-Vtc2 (Fig. 4F and G). In the *apl2*Δ mutant, the fusion protein was absent from perinuclear regions and the cell periphery, instead accumulating abnormally in patch-like structures surrounding the vacuole. These phenotypes in the *apl2*Δ strain contrasted with the WT cells, where Vtc2 was detected at perinuclear and cell surface regions and displayed a uniform distribution along the vacuolar membrane. Similar phenotypes were also detected using wide-field fluorescence in the *apm1*Δ mutant expressing mKate2-Vtc2, consistent with observations in the *apl2*Δ mutant (Fig. S7D). Dysregulation of polyP metabolism was also verified in the mutants expressing the fusion protein through visualization of polyP levels on polyacrylamide gels stained with toluidine blue O (Fig. S7E). Previous reports demonstrate that polyP supports mitochondrial function and energy metabolism in that depletion disrupts bioenergetics in mammalian cells and defects in phosphate regulation alter polyP and ATP levels in *C. neoformans* (76, 77). Considering the increased levels of polyP, we hypothesized that the AP-1 mutants would have growth defects upon inhibition of the electron transport chain complexes. Indeed, inhibitors for ETC complexes I, III, and alternative oxidase negatively impacted growth, suggesting that the AP-1 complex may indirectly affect the organelle physiology needed for polyP homeostasis (Fig. S7F).

The integrity of vacuolar functions, including polyP homeostasis, allows the cells to deal with toxicity caused by high levels of metal ions (78, 79). The observed defects in vacuolar function and altered polyP content in the mutants prompted us to test whether metal ion toxicity affects cell growth. Serial dilution assays showed that the mutants exhibited marked growth inhibition in the presence of manganese chloride or copper sulfate, but not zinc chloride, indicating a compromised ability to tolerate metal ion stress (Fig. 4H). These findings were consistent with phenotypes observed in *C. neoformans* mutants defective in late endocytic trafficking pathways (72, 80–82). Taken together, we conclude that the AP-1 complex is also crucial for vacuolar homeostasis and polyphosphate regulation, with its loss leading to polyP mislocalization and reduced tolerance to metal ion stress.

### AP-1 deficiency reduces intracellular survival within macrophages

**Fig 5:**
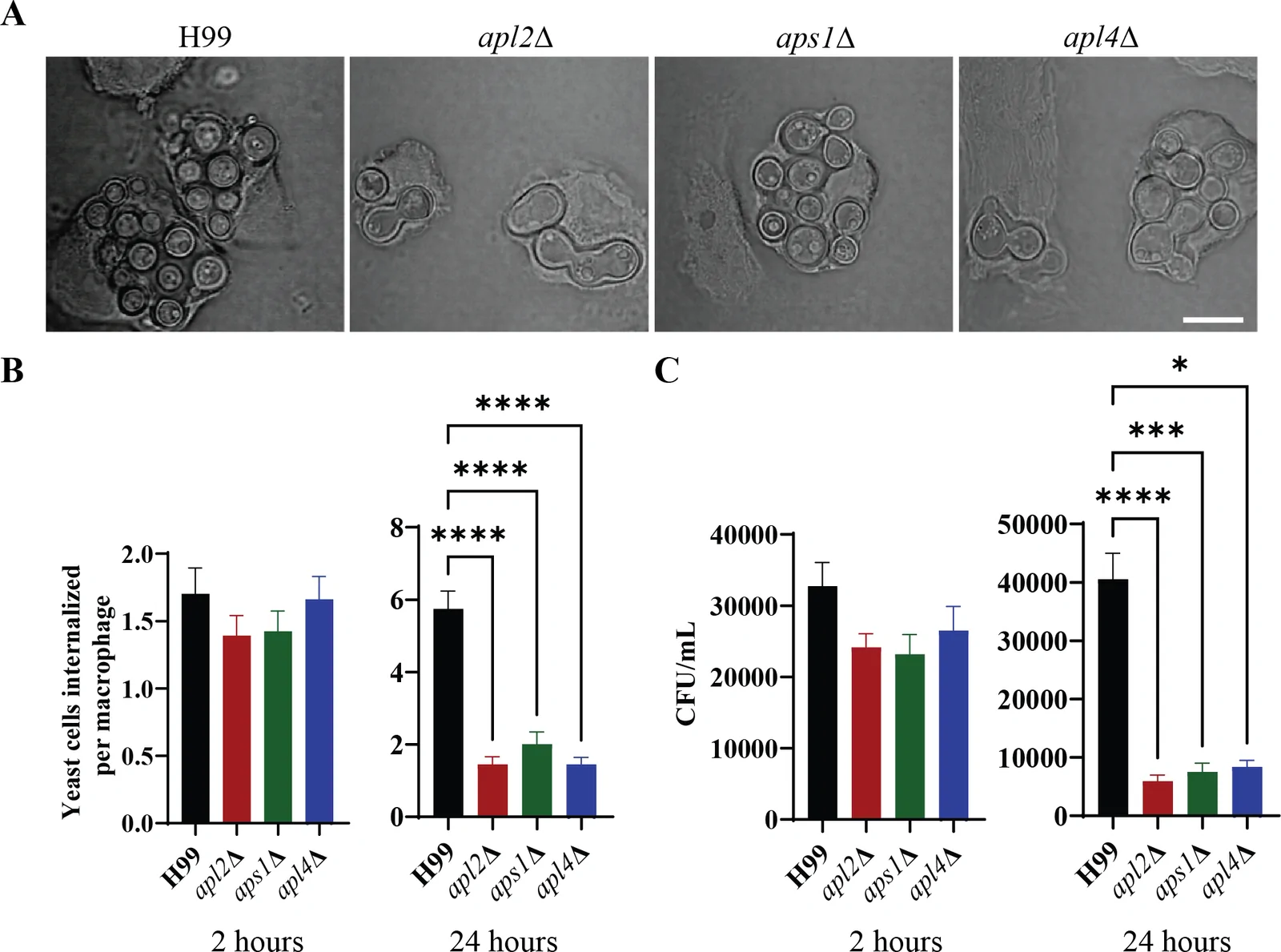
The AP-1 complex is required for intracellular survival of *C. neoformans* within macrophages *in vitro*. (**A**) Brightfield microscopy images of wild-type (H99) and single deletion mutants (*apl2*Δ, *aps1*Δ, and *apl4*Δ) inside macrophage phagolysosomes. Opsonized *C. neoformans* cells with the anti-GXM monoclonal antibody 18B7 were incubated with J774A.1 macrophages at a multiplicity of infection (MOI) of 10:1. Captured images showing macrophages fixed at 24 h post-infection. Infections were carried out at 37°C with 5% CO₂ for either 2 or 24 h. Images are representative of at least three independent experiments, each observing more than 100 macrophage cells. Scale bar (10 µm). (**B**) Quantitative assessment of yeast uptake following macrophage infection. Values indicate the mean ± SEM of the number of internalized yeasts per macrophage, determined from micrographs acquired at 2 and 24 h post-infection. (**C**) Survival assays of *C. neoformans* strains following macrophage infection at 2 and 24 h post-infection. The indicated strains were recovered from lysed macrophages, and colony-forming units (CFUs) were quantified on YPD plates after incubation at 30°C for 2 days. Data in panels **B and C** are representative of three independent experiments ± SEM. Statistical significance shown was determined by ANOVA followed by Tukey *post hoc* test (*, *P* < 0.05; ***, *P* > 0.001; ****, *P* < 0.0001).

The phenotypes of the deletion mutants suggest that loss of the AP-1 complex may alter host–pathogen interactions. We therefore conducted *in vitro* macrophage infection assays to assess intracellular survival and internalization of the mutants. Microscopic examination of infected macrophages (*n* > 50) at 24 h revealed significantly fewer internalized mutant cells compared to the WT (Fig. 5A; Fig. S8A). Notably, in contrast to WT and complemented strains, the phagocytosed AP-1 mutants displayed pronounced morphological defects similar to those observed in culture (Fig. 2A). Quantification confirmed a ~5-fold reduction in the internalization of yeasts per macrophage across all AP-1 mutants after 24 h of infection (Fig. 5B). We determined that survival of the mutants was reduced by ~8-fold relative to WT cells at 24 h post-infection (Fig. 5C; Fig. S8B), suggesting that the integrity of the AP-1 complex contributes to the viability of the phagocytosed yeasts. Because the intracellular proliferation of *C. neoformans* is linked to tolerance of the oxidative environment in phagolysosomes, we hypothesized that enhanced sensitivity to reactive oxygen species (ROS) could partially explain the reduced survival. To investigate this possibility, we performed growth assays in the presence of ROS stressors. Exposure to hydrogen peroxide (H₂O₂) or ROS inducers such as diamide and paraquat caused a slight reduction in mutants’ growth compared to the WT (Fig. S9). These results suggest that decreased sensitivity to ROS stress within the phagolysosome may only partially explain the reduced survival observed for phagocytosed yeast cells. Taken together, our findings indicate that dysfunction of the AP-1 complex compromises the ability of *C. neoformans* to persist within macrophages, a phenotype that would likely contribute to attenuated virulence *in vivo* (33).

## DISCUSSION

In this study, we explored the functional impact of a defective AP-1 complex in *C. neoformans* with deletion mutants lacking the Apl2, Apl4, Aps1, or Apm1 subunits. We discovered that the complex was required for survival of the fungus in macrophages, thus revealing potential sensitivity to killing mechanisms such as the oxidative burst (83). We found that the mutants’ sensitivity to oxidative stress contributed only partially to reduced survival, and that a combination of other phenotypes likely influenced survival. In particular, we showed that the mutants were unable to generate a WT-like polysaccharide capsule and were impaired for growth at 37°C and 39°C, phenotypes that were most notable for the mutants lacking the large subunits (Apl2 and Apl4) and less so for the mutant lacking the small subunit (Aps1).

The generation of an encapsulated yeast is a unique and defining feature of the pathogenic *C. neoformans* and *C. gattii* species (66, 84, 85). The connections between the intracellular trafficking machinery and capsule elaboration have been investigated by several groups. For example, it was demonstrated that a secretory Rab GTPase is implicated in the transport of post-Golgi exocytic vesicles containing GXM (86). Also, specific components of the endosomal sorting complex required for transport (ESCRT) machinery cooperate in the elaboration of the capsule, possibly by mediating the attachment or transport of the capsule material (11, 13, 87). Additionally, dysregulation of phosphatidylinositol 4-phosphate (PI4P) levels in mutants lacking the Sac1 phosphatase affects secretory traffic, impacting capsule production and composition (88). We hypothesized that the underlying cause for alterations in capsule development in the AP-1 mutants in our study reflects defects in secretory alterations of capsule-related proteins. Consistent with this idea, we found that AP-1 mutants lost the punctate distribution of Cas35 at specific sites on the cell surface, leading to prolonged hyperaccumulation of the protein. Interestingly, Cas35 colocalized with the ER marker Sec61 at the cell periphery, highlighting a possible connection between capsular protein foci (putative capsulosomes) and ER-PM contact sites, a structural connection suggested previously (89). Disruption of AP-1-mediated trafficking may impair the delivery of additional capsule-related proteins, indirectly exacerbating the capsule defect and promoting the abnormal accumulation of Cas35 at the cell periphery. Cas35 belongs to the Cap64-like proteins and is proposed to be involved in attaching xylose and/or O-acetyl groups to mannose in the capsule glucuronoxylomannan (GXM) chains (65, 66, 90).

The phenotypic defects of the AP-1 mutants extended to other major virulence traits including melanin production and urease secretion. These pleiotropic phenotypes are consistent with an influence on multiple pathways and processes including endomembrane trafficking and secretion. For example, melanin production in *C. neoformans* is mediated by laccases, which are transported in secretory vesicles before being secreted or deposited at the cell wall (14, 91, 92). Evidence from studies in mammalian cells indicates that the adaptor complexes AP-1 and AP-3 play major roles in regulating the sorting of tyrosinase, a melanocyte-specific protein (93, 94). Although a direct connection of the adaptor complexes regulating the transport of cargoes associated with melanin biosynthesis has not been established in *C. neoformans*, disruption of laccase transport and activity was linked to defects caused by a mutant lacking the Vps27 protein, a subunit of the ESCRT-0 complex (14). Connections between the AP-1 complex and secretion activities have been reported in a number of studies. For instance, the complex has been linked to the coordination of the formation and maturation of Weibel-Palade Bodies (WPBs), which are regulated secretory organelles in mammalian endothelial cells (95). Similar connections have been reported in the human parasites *Plasmodium falciparum* and *Toxoplasma gondii*, where AP-1 participates in the traffic, biogenesis, and maturation of rhoptry and micronemes, which are apical secretory organelles crucial for the parasitic phase (30, 31, 96, 97).

Our AP-1 mutants exhibited striking morphological abnormalities and increased sensitivity to several membrane stressors, although their sensitivity to cell wall inhibitors was not significant. Given the established roles of adaptor protein complexes in intracellular vesicular trafficking, it is reasonable to expect that their dysregulation could affect the composition and integrity of the plasma membrane and cell wall. The coordinated delivery and retrieval of cell surface components, whether membrane lipids or biosynthetic cargo, are crucial processes for maintaining cellular morphology and cell wall integrity (98–100). Coordination of secretion and endocytic pathways is fundamental in regulating fungal cell morphology, as these systems orchestrate the targeted delivery and recycling of proteins that remodel the plasma membrane and cell wall, supporting growth, polarity, and adaptation to environmental and host conditions (101–104). Early studies in *S. cerevisiae* demonstrated that AP-1 null mutants were viable and displayed no defects in growth or α-factor maturation, suggesting that while AP-1 contributes to clathrin-mediated trafficking, it is not essential under standard conditions, as other factors can compensate for its role in vesicle assembly and cargo sorting (105–108). Consistent with this, no sensitivity to temperature stress was observed in those mutants. However, *S. cerevisiae* possesses two forms of the AP-1 complex that differ in their medium subunits, conferring distinct functional properties (106). Interestingly, loss of either medium subunit in *S. cerevisiae* altered the cellular response to membrane stress, yet no major morphological changes were detected. In contrast, recent studies in the fungal species *Candida albicans*, *Aspergillus nidulans*, *Fusarium graminearum,* and *Botrytis cinerea* revealed that disruption of the AP-1 complex severely affects cellular morphology and cell wall integrity, resulting in growth defects and reduced virulence (22, 26–28). For example, in *C. albicans,* the complex was found to be indispensable for polarized exocytosis during filamentous growth but dispensable during the yeast phase (26). Overall, the major roles proposed for AP-1 in these fungi are associated with the maintenance of apical polarization during hyphal development. Our results indicate that AP-1 in *C. neoformans* is required to preserve the yeast cell shape. Taken together, these observations suggest that the functional roles of the AP-1 adaptor complex have diverged among fungal species, potentially reflecting adaptations to their distinct morphological programs and physiological requirements.

Functional compartmentalization of the five adaptor protein complexes identified in most eukaryotes has been investigated (15, 16). In the case of the mammalian AP-1, its functions have been associated mainly with TGN and endosomes (early or recycling) trafficking pathways (20). However, the presence of lysosomal hydrolase receptors in AP-1 vesicles has linked this complex with vacuolar activities. In a recent study, a unifying model has been proposed for opisthokonts where AP-1 activity mediates protein recycling processes to the Golgi from post-Golgi organelles, including the retrieval of proteins that failed to be sorted to the lysosome, assigning a more indirect role in vacuolar functions (17). In *S. cerevisiae*, loss of AP-1 function does not appear to disrupt trafficking routes to the vacuole, a process primarily mediated by the adaptor protein complex AP-3 (105, 108). This observation suggests a possible divergence in the roles of adaptor complexes in vacuolar trafficking among fungi. Nevertheless, several studies have reported or proposed direct interactions of the AP-1 complex at the vacuolar level in other organisms (68, 70). In our study, AP-1 mutants displayed pleiotropic defects indicative of compromised vacuolar function, including altered vacuolar acidification, hypersensitivity to elevated divalent cation levels, and polyphosphate overaccumulation. Collectively, these observations suggest that the AP-1 complex contributes to the regulation of protein trafficking to vacuolar compartments. It is possible that the altered vacuolar phenotypes caused by defective AP-1 mutants could be related to sorting dysregulation of SNARE trafficking within the pathways leading to pleiotropic behavior on protein localization (17, 68, 71). The vacuolar acidification defects observed in the *C. neoformans* AP-1 mutants could stem from the missorting of V-ATPase subunits or accessory proteins due to altered Golgi–endosome–vacuole trafficking. Additionally, impaired recycling of vacuolar membrane proteins or perturbations in the lipid environment may further destabilize the V-ATPase complex, ultimately compromising vacuolar homeostasis. Interestingly, linkage between the role of V-ATPases in endosomal pH and formation of clathrin coat vesicles has been reported (109).

Recently, we demonstrated that the *C. neoformans* Vam6/Vps39/TRAP1-domain protein Vam6 plays a critical role in vacuolar morphology, iron acquisition, and virulence (12). Our findings revealed that Vam6 functions within the HOPS complex to facilitate endomembrane trafficking to the vacuole (72). Notably, this includes the regulation of polyP metabolism, as evidenced by impaired trafficking of the VTC complex subunit Vtc2, which is essential for polyphosphate synthesis. Our observations on overaccumulation of polyP in the AP-1 mutants, including the altered distribution of the VTC complex, suggest that AP-1 influences polyP metabolism and may regulate the traffic of the biosynthetic machinery or the turnover enzymes such as polyphosphatases. Disruption of exo- and endopolyphosphatase activities results in an imbalance of polyphosphate metabolism, causing excessive polyP accumulation that correlates with reduced virulence in *C. neoformans* (73, 74). In *S. cerevisiae*, polyphosphate production has been associated with the activity of two VTC sub-complexes consisting of Vtc1, Vtc4, Vtc2, or Vtc3, and their subcellular localization at different compartments could be modified in response to nutritional stress (110–112). In yeast, the AP-3 adaptor complex is required for both the transport of the VTC complex accessory subunit Vtc5 to the vacuolar membrane and in the production of WT levels of polyphosphate (113). It is possible that both AP-1 and AP-3 complexes might regulate the activities of the VTC complex, coordinating the retrograde and anterograde traffic of VTC proteins at the vacuolar membrane or of enzymes involved in polyphosphate metabolism.

**Fig 6:**
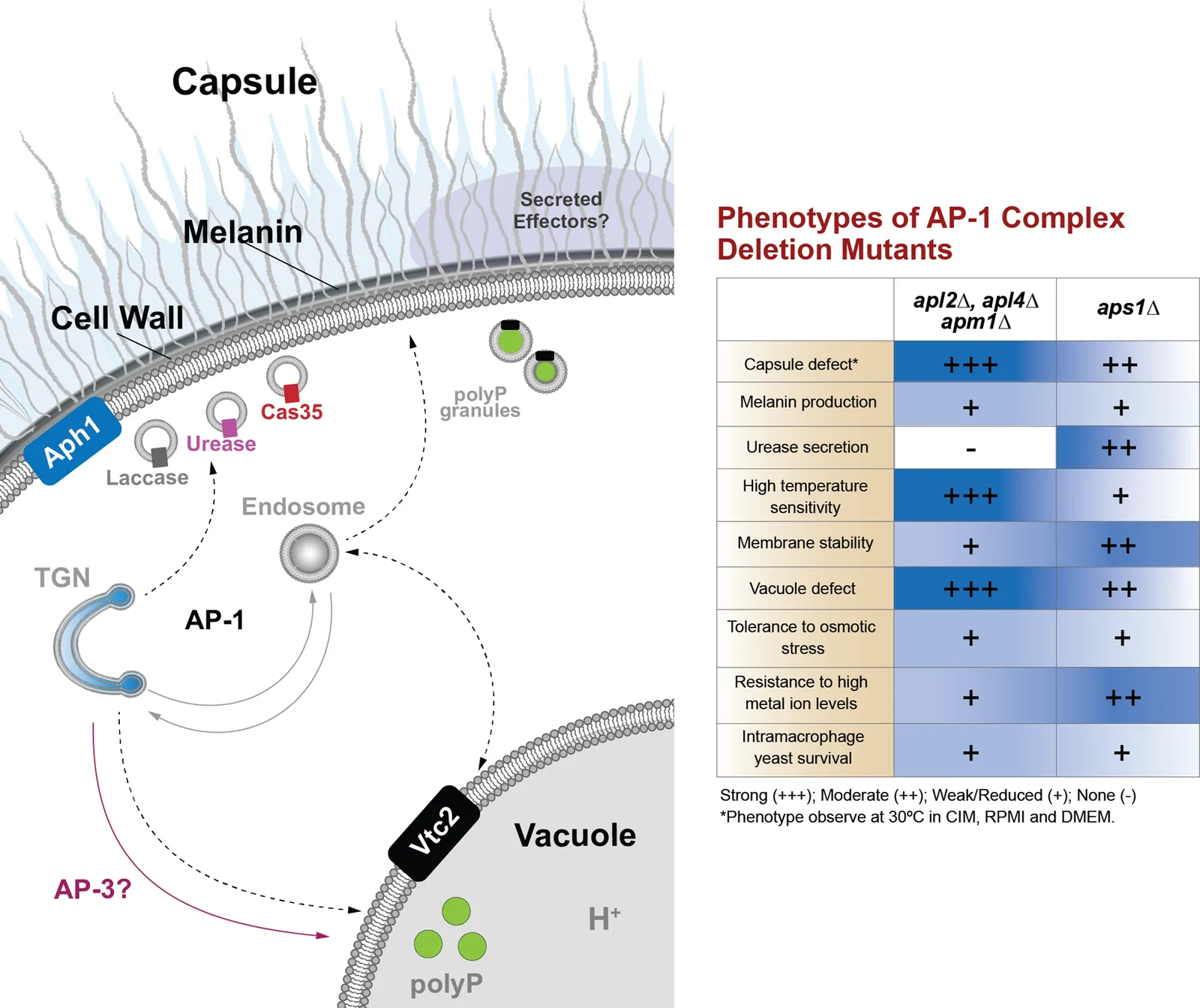
Model summarizing the role of the *C. neoformans* AP-1 in virulence-factor development and endomembrane trafficking. AP-1 participates in canonical cargo transport between the trans-Golgi network (TGN) and early/recycling endosomes (gray arrows) (32). Additional putative trafficking routes involving AP-1 subunits include transport toward the plasma membrane and the vacuole, originating either from the TGN or from endocytic vesicles (dashed black arrows). In *C. neoformans*, the AP-1 complex is required for the formation of major virulence factors—including the polysaccharide capsule, melanin, and urease secretion—as well as for maintaining cellular homeostasis during stress. These functions collectively contribute to the organism’s ability to cause disease (33). Loss of AP-1 subunits results in multiple phenotypic defects (see summary table for phenotypes of each AP-1 subunit mutant), including reduced and structurally altered capsule, diminished melanin deposition/production, abolished urease secretion, and broader physiological abnormalities. These defects indirectly suggest impaired delivery of secretory cargo (e.g., Aph1, urease, laccases, Cas35, polyP) to the cell surface or extracellular space. Defective vacuolar functions in AP-1 mutants, together with disrupted polyP homeostasis and abnormal localization of the Vtc2 subunit of the polyP-complex, further support a role for AP-1 in late endomembrane-trafficking steps.

In summary, the absence of the AP-1 subunits caused pleiotropic phenotypes in *C. neoformans*, including defects in the elaboration of major virulence factors (Fig. 6). Thus, the integrity of the complex is likely relevant for the ability of the fungus to cause disease, a conclusion supported by the survival defect upon phagocytosis and by the results of our virulence assays in a mouse model of cryptococcosis (33).

## MATERIALS AND METHODS

### Strains, plasmids, and media

*Cryptococcus neoformans* var. *grubii* H99 (serotype A) served as the WT strain in this study. All fluorescently tagged and deletion mutant strains are listed in Table S1. Maintenance of the strains, propagation, and serial dilution growth assays were performed in YPD rich agar medium (1% yeast extract, 2% Bacto‐peptone, 2% D‐glucose, and 2% agar). Agar plates for growth assays in minimal media were prepared with yeast nitrogen base (YNB, DIFCO) pH 5.6 or adjusted to pH 7.2 with 1M HEPES. Capsule production was induced using capsule inducing media (CIM), also referred to as defined low-iron media, as described previously (114). Briefly, the following components were dissolved in low iron water chelated with resin Chelex 100 and adjusted to pH 7 with MOPS (1M): (5 g L^−1^ glucose, 5 g L^−1^ L‐asparagine, 0.4 g L^−1^ K_2_HPO_4_, 0.25 g L^−1^ CaCl_2_·2H_2_O, 0.08 g L^−1^ MgSO_4_·7H_2_O, 4.78 g L^−1^ HEPES, 1.85 g L^−1^ NaHCO_3_, 1 mL of 1,000× salt solution (0.005 g L^−1^ CuSO_4_·5H_2_O, 2 g L^−1^ ZnSO_4_·7H_2_O, 0.01 g L^−1^ MnCl_2_·4H_2_O, 0.46 g L^−1^ sodium molybdate, and 0.057 g L^−1^ boric acid). After filter sterilization, the medium was supplemented with 0.4 mg mL^−1^ sterile thiamine and salt solution. All chemicals were from Sigma-Aldrich (St. Louis, MO) unless otherwise stated. For host-inducing conditions, experiments were performed using RPMI 1640 and Dubelcco’s Modified Eagle (DMEM) (Gibco, USA), each supplemented with heat-inactivated fetal bovine serum (FBS; Thermo Fisher Scientific, Waltham, MA, USA).

### Construction of gene deletion strains

The single gene deletion mutants *aps1*Δ, *apl2*Δ, and *apl4*Δ were generated using strains (designated -hm) from the *C. neoformans* (KN99) knockout collection library obtained from the Fungal Genetics Stock Center (115, 116). Briefly, deletion cassettes were PCR amplified from gDNA of the *aps1*Δ-hm, *apl2*Δ-hm, and *apl4*Δ-hm deletion mutants with primer pairs ES (412, 415), ES (472, 473), and ES (466, 468), respectively (Table S2). Purified cassettes containing the nourseothricin-resistance marker (NAT; nourseothricin acetyl transferase) were introduced into *C. neoformans* (H99) to obtain at least two independent mutants using biolistic transformation, as described previously (117). The same NAT cassettes for deletion of *APS1* or *APL2* were employed to delete the genes in strains expressing Aph1-DsRed, mCherry-Cas35, or mKate2-Vtc2. Independent single *apm1*∆ deletion mutants designated *apm1*Δ-3a and *apm1*Δ-6b were generated with a cassette containing the hygromycin-resistance marker (HYG) using the split-marker method for targeted gene deletion (118). The deletion cassette was introduced into *C. neoformans* (H99) as previously mentioned. Briefly, the genomic 5′ and 3′ flanking regions, including untranslated and partial coding sequences of the *APM1* gene, were obtained using primer pairs ES (646, 647) and ES (648, 649), respectively. The HYG cassette was obtained from plasmid pESL018-2 (114) using primer pairs ES (481, 482). Recombinant DNA split-marker fragments were generated by fusing each flanking fragment 5′ and 3′ with a truncated hygromycin cassette using primers ES (646, 634) and ES (649, 635), respectively. HYG deletion cassette insertions in the *apm1*Δ single mutants were PCR-screened with primers ES (650, 651). The presence or absence of the genes *APS1* (CNAG_02113), *APL2* (CNAG_05248), *APL4* (CNAG_07318), and *APM1* (CNAG_03317) in the deletion and complemented mutants was verified by PCR using primer pairs ES (423, 424), ES (469, 470), ES (466, 467), and ES (667, 647), respectively.

Complementation of the single deletion mutants *aps1*Δ and *apl2*Δ was achieved using the safe-haven plasmids listed in Table S1. Briefly, primer pairs ES (476, 477) and ES (483, 484) were used to PCR amplify the endogenous promoters and sequences for both *APS1* and *APL2*, respectively. The corresponding DNA fragments were cloned into plasmid pESL018-2 amplified with primers ES (478, 479), resulting in the complementing plasmids pESL018-2-pAPS1-Aps1 and pESL018-2-pAPL2-Apl2. We also generated complementation vectors using the elongation factor promoter EF1 for the expression of *APS1* and *APM1* genes. Briefly, primer pairs ES (446, 477) and ES (667, 668) were used to amplify the gene sequences of *APS1* and *APM1*, respectively. The corresponding DNA fragments were cloned into plasmids pESL018-2 and pESL018-9, PCR amplified with primers ES (480, 478) and ES (161, 478), resulting in complementing vectors pESL018-2-pEF1-Aps1 and pESL018-9-pEF1-Apm1. Vectors were introduced into the corresponding deletion mutant strains. The presence or absence of the *APS1*, *APL2*, and *APM1* genes in the complemented deletion mutant strains was determined using primer pairs ES (423-ES424), ES (487, 488), and ES (647, 667), respectively. Screening of mutants and verification of the integration of complemented vectors at safe genomic locations was determined by multiplex PCR analysis using primers UQ2962, UQ2963, UQ1768, and UQ3348, as previously described (119).

### Sample preparation and SEM

*C. neoformans* yeast cells were cultured in CIM for 48 h at 30°C, then washed twice with 1× PBS (pH 7.4) and fixed for 1 h in 2.5% glutaraldehyde in 0.1 M sodium cacodylate buffer (pH 7.4) at room temperature. After fixation, the cells were washed twice with 1× PBS. A 20 μL aliquot of the fixed cell suspension was placed onto poly-L-lysine-coated coverslips (BioCoat Ref. 354085). Samples were sequentially dehydrated in ethanol at concentrations of 50%, 60%, 70%, 80%, 90%, and then three 100% ethanol washes. Dehydrated samples were critical-point dried using a Tousimis Samdri-795 dryer and coated with 10 nm of iridium using a Leica EM MED020 sputter coater. Imaging was performed on a Helios NanoLab 650 dual-beam scanning electron microscope (Thermo Fisher, MA) equipped with an Everhart-Thornley detector, operating at a voltage range of 10–15 kV and a working distance of 4 mm.

### Assessment of virulence factor production

Capsule formation was assessed using DIC microscopy with a Zeiss Plan-Apochromat 100×/1.46 oil objective on a Zeiss Axioplan 2 microscope, coupled with a CMOS camera (ORCA-Flash4.0 LT; Hamamatsu Photonics). Cells (1 × 10^3^ cells/mL) were incubated in CIM (3–5 mL) in the dark for 24–72 h at 200 rpm and 30°C or 37℃ and stained with India ink to visualize the capsule. To evaluate capsule production under host-like conditions, cells at the same concentration were cultured in RPMI or DMEM at 37°C with 5% CO₂ for 24–96 h and visualized using the same staining procedure. Melanin production was assessed by culturing overnight-grown cells (1 × 10³ cells/mL) in 5 mL of defined liquid medium containing 0.1% glucose and supplemented with L-3,4-dihydroxyphenylalanine (L-DOPA), as previously described (44). For urease secretion assays, cells (2 × 10³ cells/mL) were grown in 1 mL of Christensen’s urea broth for 18 h at 30°C or 37°C. For both assays, cell densities were normalized prior to collection, and culture supernatants were analyzed spectrophotometrically at 405 nm and 570 nm to quantify melanin and urease activity, respectively.

### Flow cytometry analysis of membrane permeability

Membrane permeability was assessed using a CytoFLEX S Flow Cytometer (Beckman Coulter) equipped with four laser lines (405, 488, 561, and 633 nm) fitted with a phycoerythrin (PE) filter (585/42). For each sample, 30,000–40,000 cells were analyzed. Cells were grown in YPD medium for 16 h at 30°C and ~200 rpm, washed twice with dH₂O or PBS, and normalized to 1 × 10⁸ cells/mL. Cells (1 × 10^7^ cells/mL) were then incubated in YPD with or without 0.05% SDS for 3 h under identical conditions. After treatment, cells were washed three times with PBS and stained with propidium iodide (2.5 μg/mL) for 30 min at 30°C with shaking (~200 rpm). Stained cells were then washed twice with PBS and kept on ice prior to analysis. Flow cytometry data were processed using FlowJo v10.8 (BD Life Sciences) and CytExpert v2.4.0.28 (Beckman Coulter). The gating strategy is shown in Fig. S5B. Statistical analyses were performed using GraphPad Prism.

### Fluorescence microscopy analysis

Wide-field fluorescence microscopy was conducted using a Zeiss Axio Imager.M2 microscope, as described above. Laser scanning confocal micrographs were obtained using an inverted Axio Observer Z1/7 microscope and a Plan-Apochromat 63×/1.40 oil DIC M27 objective lens. The Airyscan 2 detector was operated in super-resolution mode, with emission detection ranges of 422–497/607–735 nm for mKATE2 and 420–480/495–550 nm for SF, GFP, or quinacrine, using laser excitation lines at 561 nm and 488 nm, respectively. For visualization of polyphosphate (polyP) granules, nuclei, and membranes, cells were stained with a high concentration of DAPI and the lipophilic dye FM4-64, as previously described (72). Briefly, cells were grown overnight in YPD at 30°C, washed three times with PBS, and stained with DAPI (100 μg/mL) for 30 min at room temperature. Following staining, cells were washed three additional times with PBS. Fluorescence images were acquired using BrightLine full multiband filter sets (Ex/Em 407/530 nm) to visualize polyP, DAPI filter sets (Ex/Em 359/461 nm) to detect DNA, and Texas Red filter sets (Ex/Em 592/614 nm) for membrane detection. For visualization of fluorescently tagged proteins, cells expressing fusion constructs containing mKate2, DsRed, or mCherry were imaged using a filter set (Ex/Em 587/610 nm), whereas GFP-tagged proteins were visualized with an eGFP filter set (Ex/Em 488/509 nm), as previously described (72). For fluorescence quantification, mCherry-Cas35 signal intensities were measured at the cell periphery within manually defined regions of interest (0.95 µm²). For cell wall visualization, cells were stained with Solophenyl Flavine 7GFE (SF, 0.01%) and imaged using the eGFP filter set, as described previously. Vacuoles were visualized using the weak base quinacrine, which selectively accumulates in acidic compartments. Briefly, cells were washed once with HG3 buffer (100 mM HEPES/KOH, 3% glucose, pH 7.5), stained with 200 μM quinacrine dihydrochloride for 10 min, and incubated on ice for 5 min. Subsequently, cells were washed twice and resuspended in HG3 buffer before imaging with fluorescence filter sets (Ex = 470/40 nm; Em = 525/50 nm).

### Stress and drug response assays

Exponentially growing WT and mutant strains were washed and adjusted to an initial density of 1 × 10⁸ cells/mL in dH₂O. Tenfold serial dilutions were prepared, and 5 μL of each dilution was spotted onto YPD agar plates supplemented with the indicated stress-inducing or inhibitory compound. Growth analysis upon osmotic stress was examined using sodium chloride (NaCl, 1.5 mM) or potassium chloride (KCl, 1.5 mM). To assess sensitivity to membrane integrity and analyze the growth response of the strains to azole antifungal agents, SDS (0.01%), fluconazole (10 µg mL^−1^), and miconazole (0.01 µg mL^−1^) were used, respectively. To evaluate secretory and AP-1 complex-specific inhibition, cells were exposed to brefeldin A (BFA, 30 µg/mL) and the membrane traffic inhibitor A5 (50–100 µM), respectively. To test sensitivity to high levels of divalent metal ions, strains were grown in YPD with zinc chloride (ZnCl_2_, 2.5 mM), copper sulfate (CuSO_4_, 10 mM), or manganese chloride (MnCl_2_ ·4H_2_O, 3.75 mM). For assessing vacuole function, YPD was supplemented with the following compounds: bafilomycin A (1 µM), concanamycin A (500 nM), chloroquine (6 mM), or quinacrine (1.6 mM). To assess the response to ROS, the strains were exposed to hydrogen peroxide (H_2_O_2_, 3 mM), diamide (1 mM), or paraquat (0.7 mM). The sensitivity to cell wall inhibitors was tested using caffeine (CAF, 1–1.5 mg/mL) and calcofluor white (CFW, 0.375–3 mg/mL). Plates or liquid assays were incubated at 30°C, 37°C, or 39°C for 1–3 days before being imaged to assess growth phenotypes.

### Construction of *C. neoformans* strains expressing mKATE2

Strains expressing red fluorescent mKate2–tagged proteins were generated as follows. The plasmid pESL018-3, a modified version of the *C. neoformans* heme sensor (CnHS) vector pESL018-2 (72) containing a neomycin resistance cassette and a safe-haven locus sequence, was further engineered to remove the GFP-based heme-sensing domain while retaining the red fluorescent marker mKate2, resulting in vector pESL018-9. We inserted the N-terminal HA-tag into mKate2 using the primer pair (ES 573, 574), resulting in vector pESL018-9-HA-mKate2. Subsequently, we cloned the *APL2* gene obtained with primer pair (ES 692, 693) into the vector amplified with the primer pair (ES 161, 641), yielding pESL018-9-Apl2-HA-mKate2. The expression of the tagged protein was driven by the elongation factor 1 promoter (EF1). The Apl2-HA-mKate2 construct was introduced into *apl2*Δ mutant to validate the functionality of the fusion protein via phenotypic complementation and to further perform microscopy analysis. Fluorescence microscopy images were acquired using the equipment described above and processed with ZEN Lite 2.3 (version 2.3.69.1000; Carl Zeiss) and ImageJ 1.53q for image analysis.

### Macrophage uptake and survival assays

J774A.1 murine macrophage-like cells (ATCC, Manassas, Virginia) were cultured in high-glucose DMEM (Fisher Scientific) supplemented with 10% heat-inactivated FBS, 2 mM L-glutamine (Gibco), and maintained for no more than 20 passages post-thawing from liquid nitrogen. To assess phagocytosis and intracellular survival, J774A.1 cells were seeded into 24-well plates and stimulated with 150 ng/mL phorbol 12-myristate 13-acetate (PMA) for 1 h prior to infection. WT and mutant cells were opsonized for 1 h with the anti-GXM monoclonal antibody 18B7 and then added to macrophages at an MOI of 10:1 (yeast:macrophage). Infections were carried out at 37°C with 5% CO₂ for either 2 or 24 h. Following infection, non-internalized yeast cells were removed by washing three times with 1× PBS. To quantify internalized yeast, macrophages were lysed with sterile dH₂O, and lysates were plated on YPD agar to determine CFUs at both time points. For imaging-based analysis, J774A.1 cells were seeded onto 8-well chamber slides in DMEM and allowed to adhere overnight. The following day, cells were washed and maintained in serum-free DMEM during infection with opsonized yeast. DIC microscopy was used to capture live-cell images 2 h post-infection for phagocytosis assessment, with at least 300 macrophages analyzed per strain in each experiment. For enhanced visualization of yeast cell morphology, samples were stained with Solophenyl Flavine 7GFE (SF), as described above. For intracellular survival analysis, DIC images were acquired 24 h post-infection after removal of extracellular yeast by PBS washing.

### Assessment of polyP accumulation

Intracellular polyP levels were measured as previously described (72). Briefly, cells were grown overnight in YPD at 30°C. RNA was extracted using a citrate buffer, and cells were disrupted in a bead mill to release both RNA and polyphosphate. A total of 10–15 μg of RNA was then loaded onto a native polyacrylamide gel and electrophoresed in 1× Tris-borate-EDTA (TBE) buffer. Following electrophoresis, the RNA and polyphosphate were fixed using acetate, stained with toluidine blue O, and destained in acetate according to established protocols. Polyphosphate type 45 and/or 700 (P700; Kerafast, Boston, MA) at 10 μg served as molecular weight marker controls. Densitometric analysis was carried out using ImageJ software. Gel images were imported, and the regions corresponding to polyP (beneath the RNA band and extending to the gel edge) were selected and quantified. Background signal was measured from a blank gel region and subtracted from each sample. The resulting values were normalized to the WT or untreated control, which was set to a value of 1. Experiments were performed in biological triplicate, and representative data are presented. The accumulation of polyP granules was evaluated by fluorescence microscopy of DAPI-stained cells (100 μg/mL) imaged with the BrightLine filter set (Ex/Em 407/530 nm).

### Liquid growth assays in host-like conditions

Overnight YPD cultures were inoculated into RPMI or DMEM at an initial density of 0.1–0.2 OD_600_. Cultures were dispensed into flat-bottom 48-well plates containing media supplemented with heat-inactivated FBS and incubated in the dark at 37°C with 5% CO₂ for 3 days without shaking. Absorbance (OD_600_) was measured every 24 h using an Agilent BioTek Synergy H1 Microplate Reader.

### Statistical analysis

Statistical analyses were conducted using unpaired Student’s *t*-tests, Kruskal-Wallis test, Mann-Whitney test, or one-way ANOVA, as appropriate. All statistical procedures were performed using GraphPad Prism software.

## SUPPLEMENTAL MATERIAL

Supplemental material iai.00098-26-s0001.pdf Fig. S1 to S9; Tables S1 and S2.

ASM does not own the copyrights to Supplemental Material that may be linked to, or accessed through, an article. The authors have granted ASM a non-exclusive, world-wide license to publish the Supplemental Material files. Please contact the corresponding author directly for reuse.
