# Supplementary material for: The AP-1 adaptor complex is required for cell surface modifications and the survival of Cryptococcus neoformans in phagocytic cells
Source: Infect Immun. 2026 Aug 4;94(9):e00098-26. doi: 10.1128/iai.00098-26 (PMC13552115; doi:10.1128/iai.00098-26)
Supplement: Supplementary file 1 [file iai.00098-26-s0001.pdf]

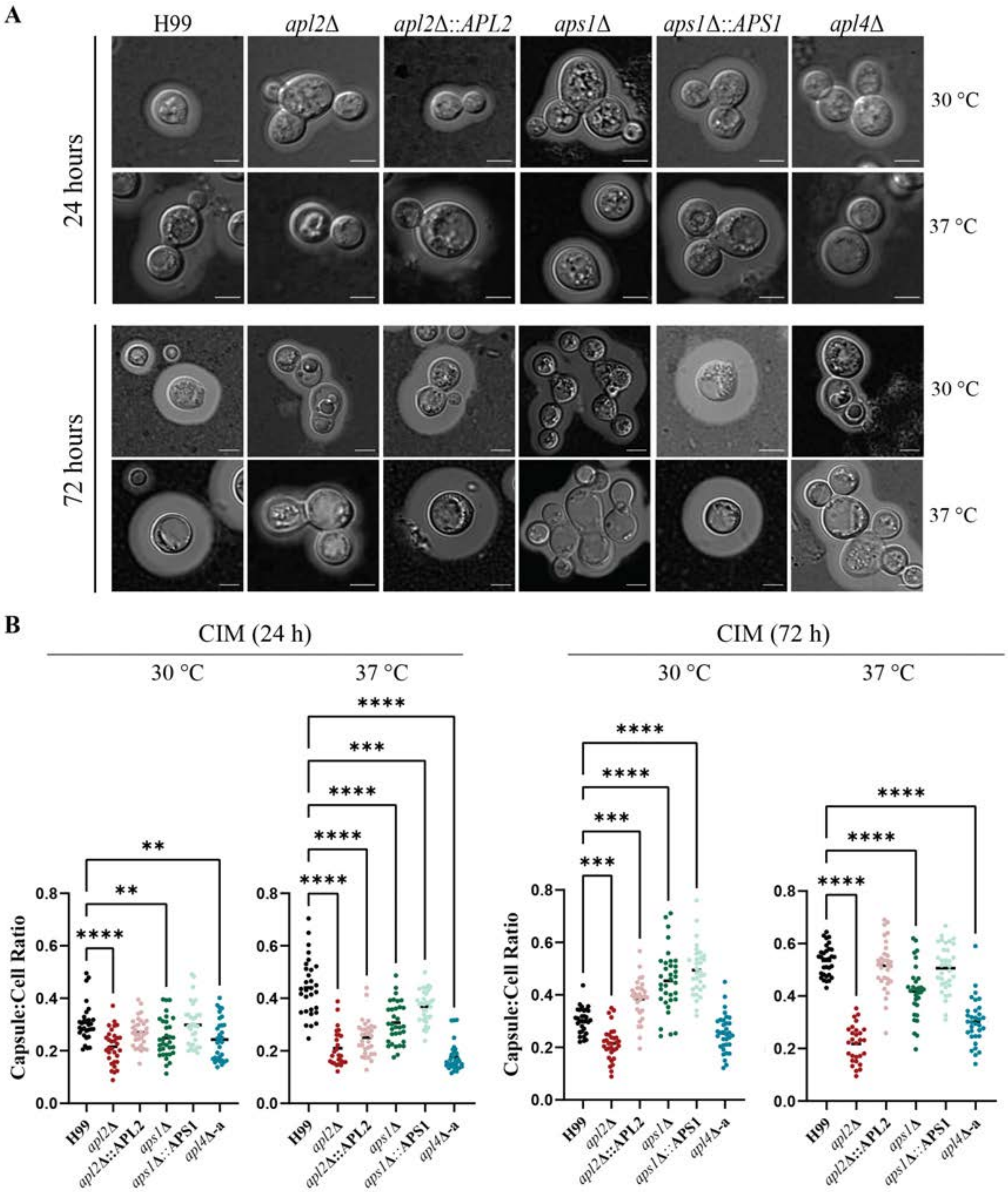

Fig S1. Loss of adaptor protein 1 (AP-1) subunits affects capsule production. (A) Differential interference contrast (DIC) microscopy of wild-type (H99), single deletion mutants (*apl2*Δ, *aps1*Δ, *apl4*Δ-4a), and complemented strains (*apl2*Δ::APL2, *aps1*Δ::APS1). Cells (1×10^3 cells/mL) were cultured in Capsule Inducing Medium (CIM) for 24 and 72 hours at 30 °C or 37 °C and stained with India ink to visualize the polysaccharide capsule. Images are representative of at least three independent experiments, each observing more than 30 cells. Scale bars (5 µm). (B) Capsule thickness–to–Cell diameter ratios for each strain were obtained from cells (in A) grown under capsule inducing conditions. Results for each condition and time point are representative of three independent experiments and include individual measurements ($n \geq 30$) as well as the mean. Statistical significance shown was determined by ANOVA followed by Dunnett *post hoc* test (**, P> 0.01; ***, P> 0.001; ****, P < 0.0001).

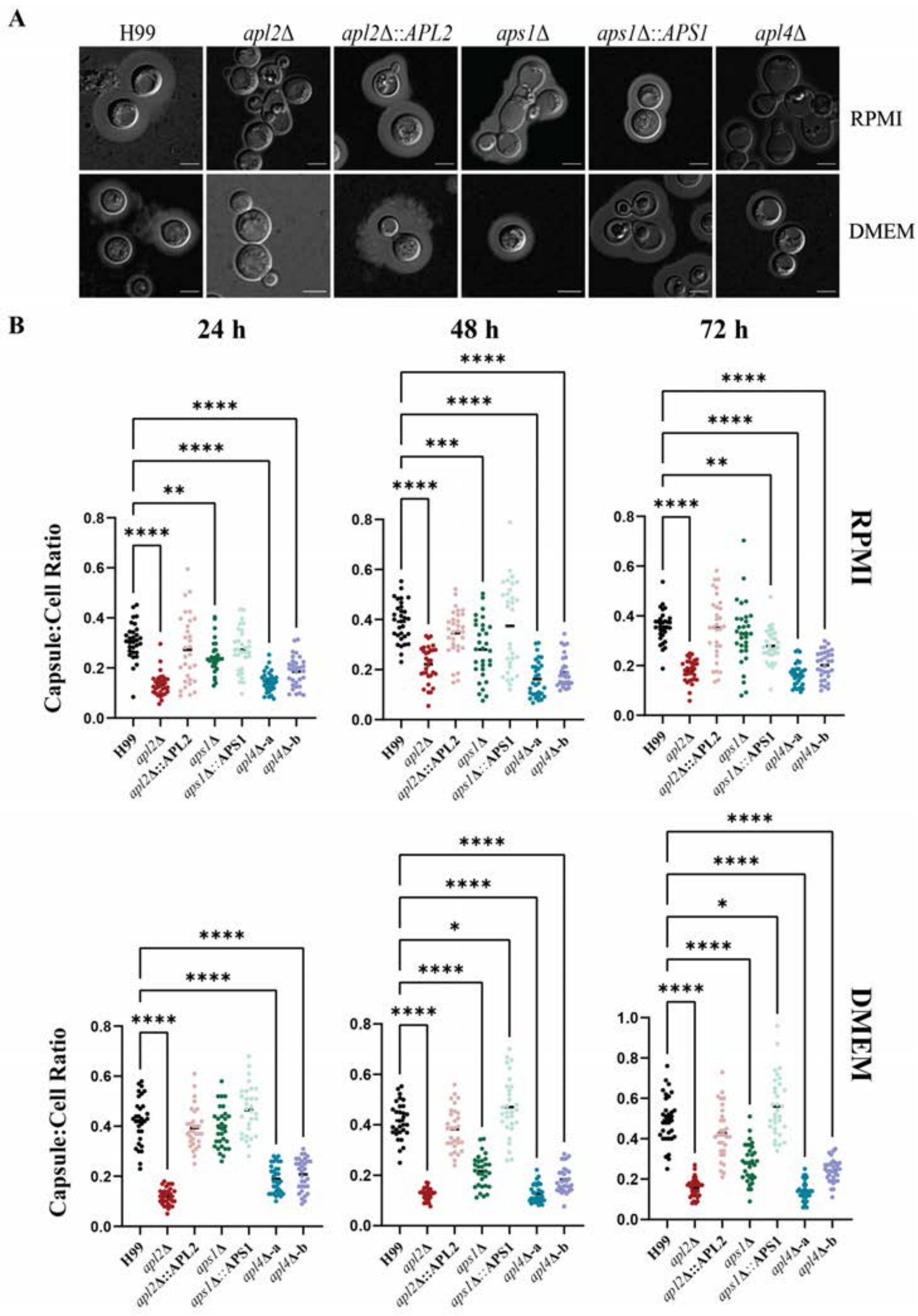

Fig S2. Host-like conditions mimic the capsule defects observed in strains impaired in AP-1 complex grown in CIM (Capsule Inducing Medium). (A) Differential interference contrast (DIC) microscopy of wild-type (H99), single deletion mutants (*apl2*Δ, *aps1*Δ, *apl4*Δ-4a), and complemented strains (*apl2*Δ::APL2, *aps1*Δ::APS1). Cells (1×10^3 cells/mL) were cultured in the host–like conditions: RPMI and DMEM for 24, 48 and 72 hours at 37 °C with 5% CO_2. Cells were stained with India ink to visualize the polysaccharide capsule. Images are representative of two independent experiments, each observing 30 cells. Scale bars (5 µm). (B) Capsule thickness–to–Cell diameter ratios for each strain were obtained from cells grown under host-like conditions as previously mentioned. Results for each condition and time point are representative of two independent experiments and include individual measurements ($n \geq 30$) as well as the mean. Statistical significance shown was determined by ANOVA followed by Dunnett *post hoc* test (*, P> 0.05; **, P> 0.01; ***, P> 0.001; ****, P < 0.0001).

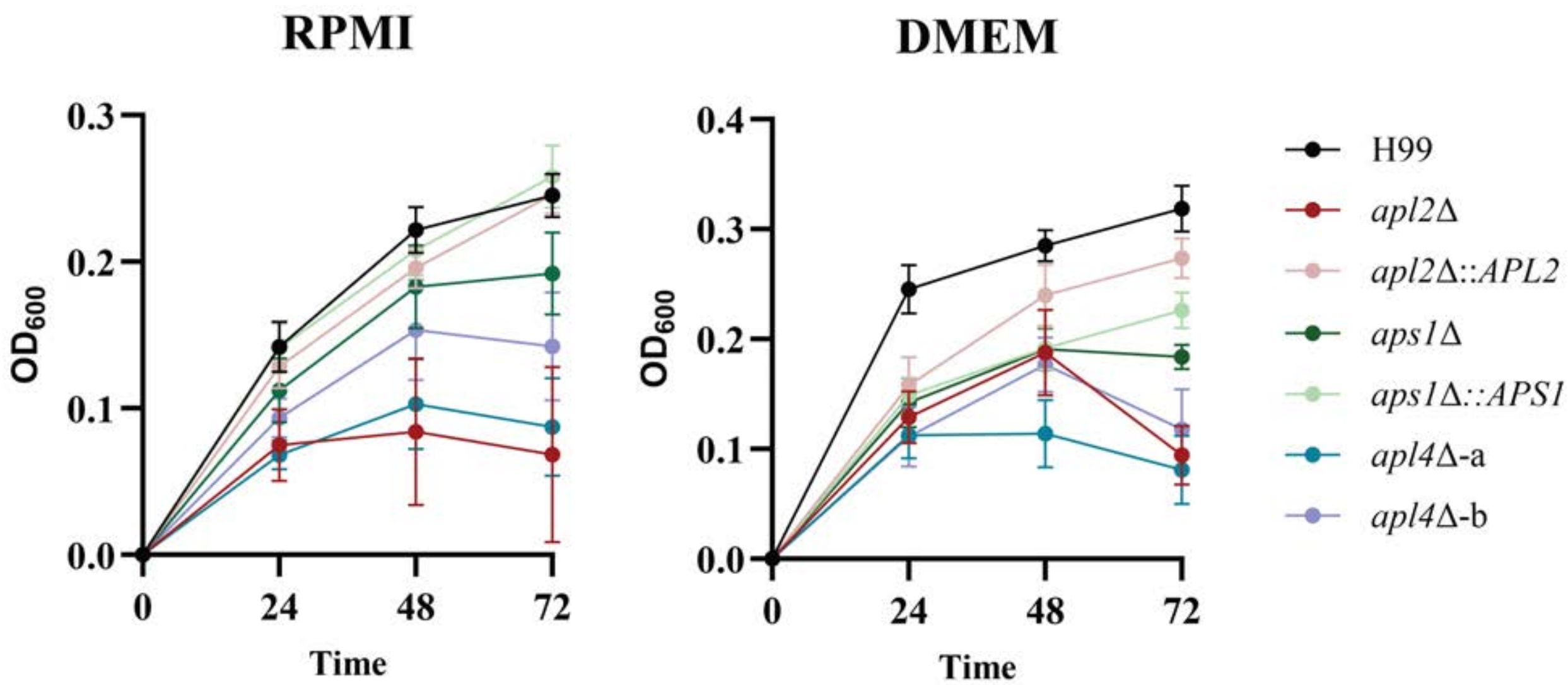

Fig S3. Impaired AP-1 complex function impacts cell growth under host-like conditions. Indicated strains were grown in YPD rich medium and subsequently inoculated at 0.1–0.2 OD_{600} in RPMI and DMEM. Cultures were incubated for 24, 48 and 72 hours at 37°C with 5% CO_2. Absorbance (OD 600 nm) was measured every 24 hours. Data for each condition and time point are representative of at least three technical replicates and two independent experiments.

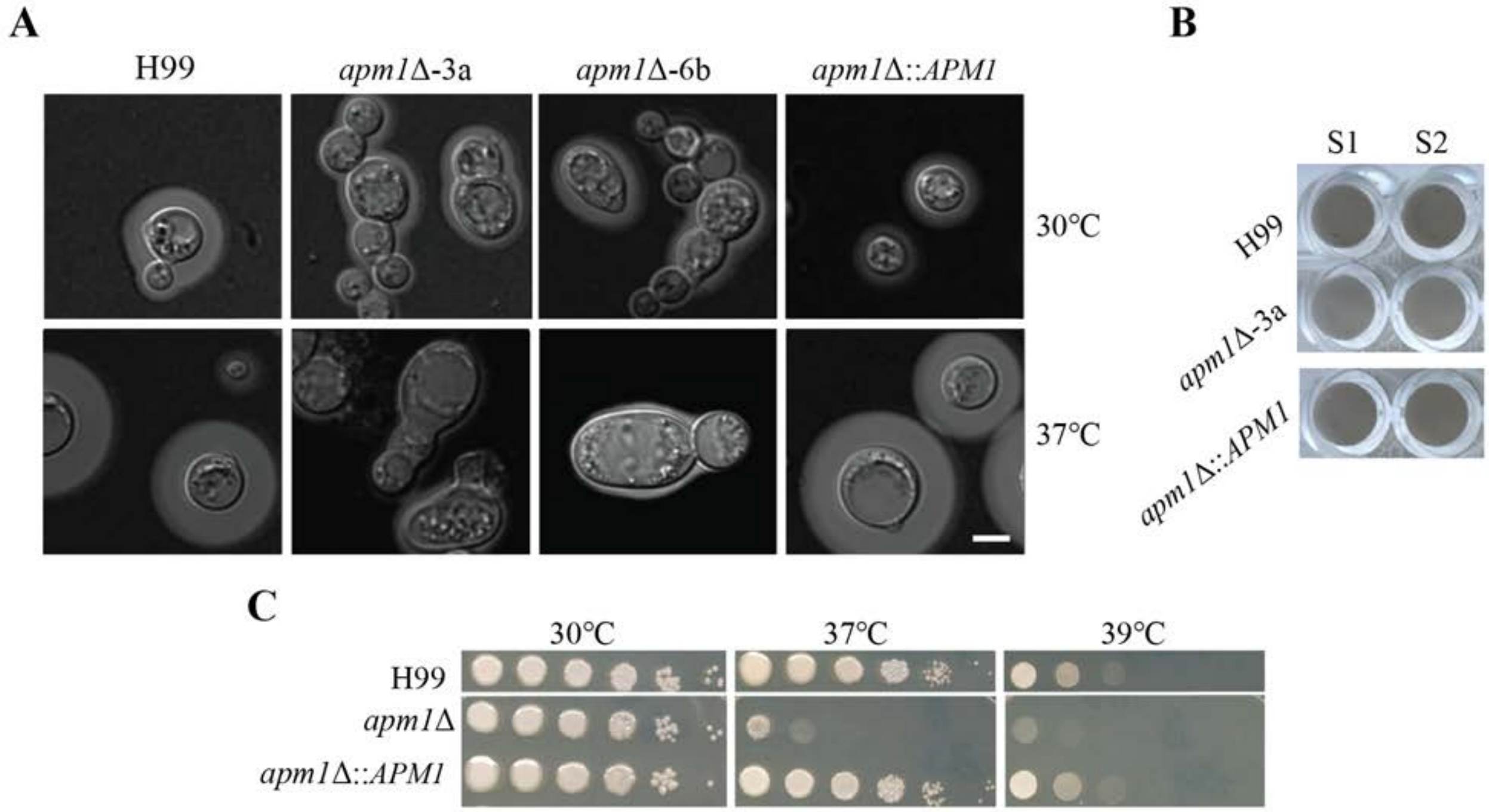

Fig S4. Loss of the medium subunit (Apm1) in adaptor protein 1 (AP-1) influences virulence factor development. (A) Differential interference contrast (DIC) microscopy of wild-type (H99), single deletion mutants (*apm1*Δ-3a and *apm1*Δ-6b), and complemented strain (*apm1*Δ::*APM1)*. Cells (1×10^3 cells/mL) were cultured in defined low-iron medium (CIM) for 48 hours at 30 °C or 37 °C and stained with India ink to visualize the polysaccharide capsule. Scale bar, 5 µm. (B) Melanin production in two samples (S1 and S2) assessed from cells cultured overnight in defined L-DOPA liquid medium. (C) Tenfold serial dilutions of the cells were spotted onto solid YPD medium and incubated at different temperatures for 2–3 days. Plates were then scanned, and representative images from at least three independent experiments are shown. Note that panels B and C were spliced for labeling purposes.

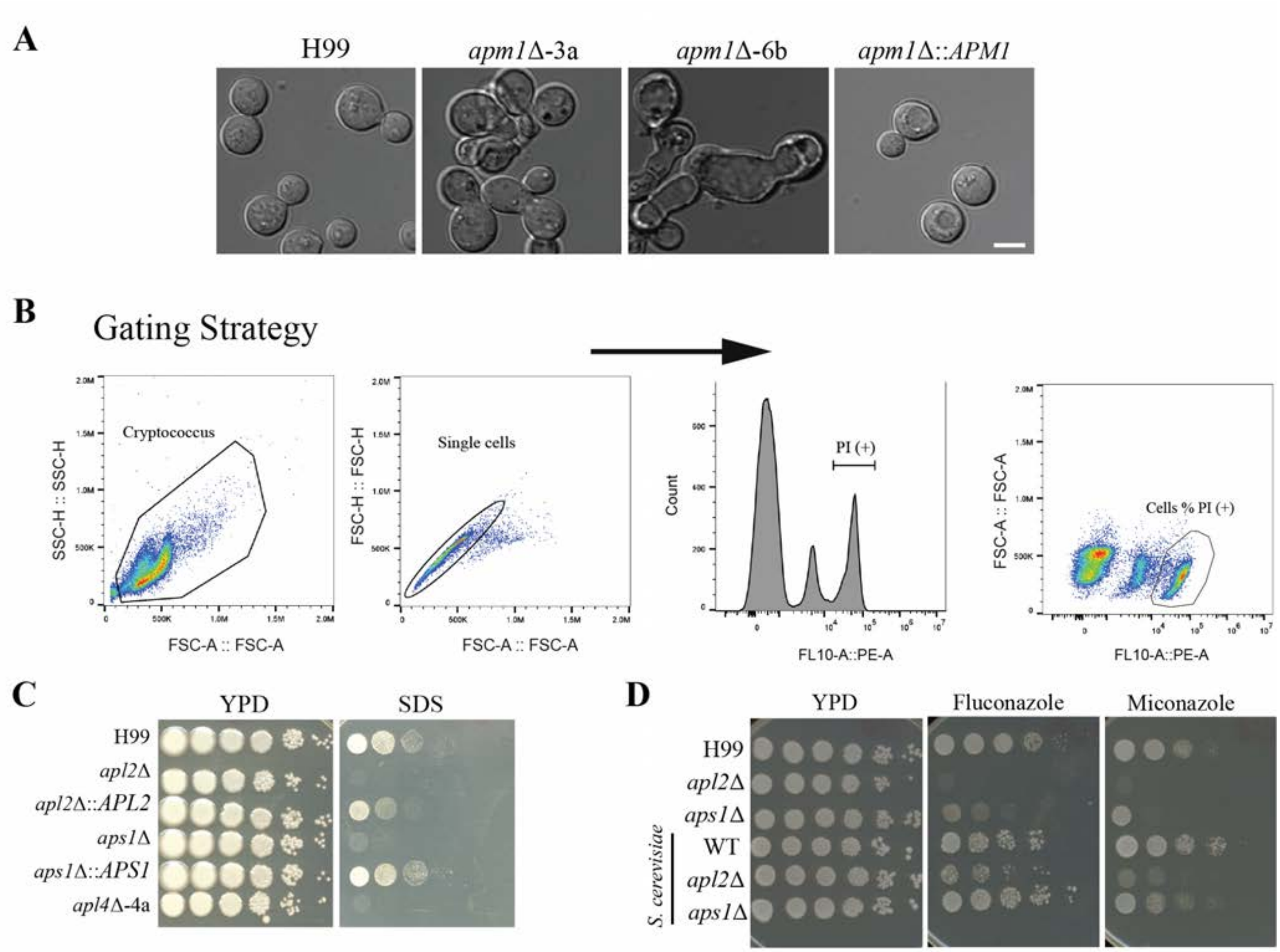

Fig S5. Disruption of the AP-1 complex impact cellular morphology and enhances sensitivity to cell membrane stress. (A) Differential interference contrast (DIC) microscopy of wild-type (H99), single deletion mutants (*apm1*Δ-3a and *apm1*Δ-6b), and complemented strain (*apm1*Δ::*APM1)*. Cells were cultured in YPD medium overnight at 30 °C. Images are representative of at least three independent experiments, each observing more than 100 cells. Scale bar (5 μm). (B) Gating strategy for flow cytometry experiments performed in *C. neoformans* cells stained for cell permeability with propidium iodide (PI). (C, D) Tenfold serial dilutions of the indicated strains were spotted onto solid YPD medium supplemented with or without SDS (0.05%) or the azole antifungals fluconazole (10 μg/mL) and miconazole (0.01 μg/mL). Plates were incubated at 30 °C for 2–3 days before being scanned. Representative images from at least three independent experiments are shown.

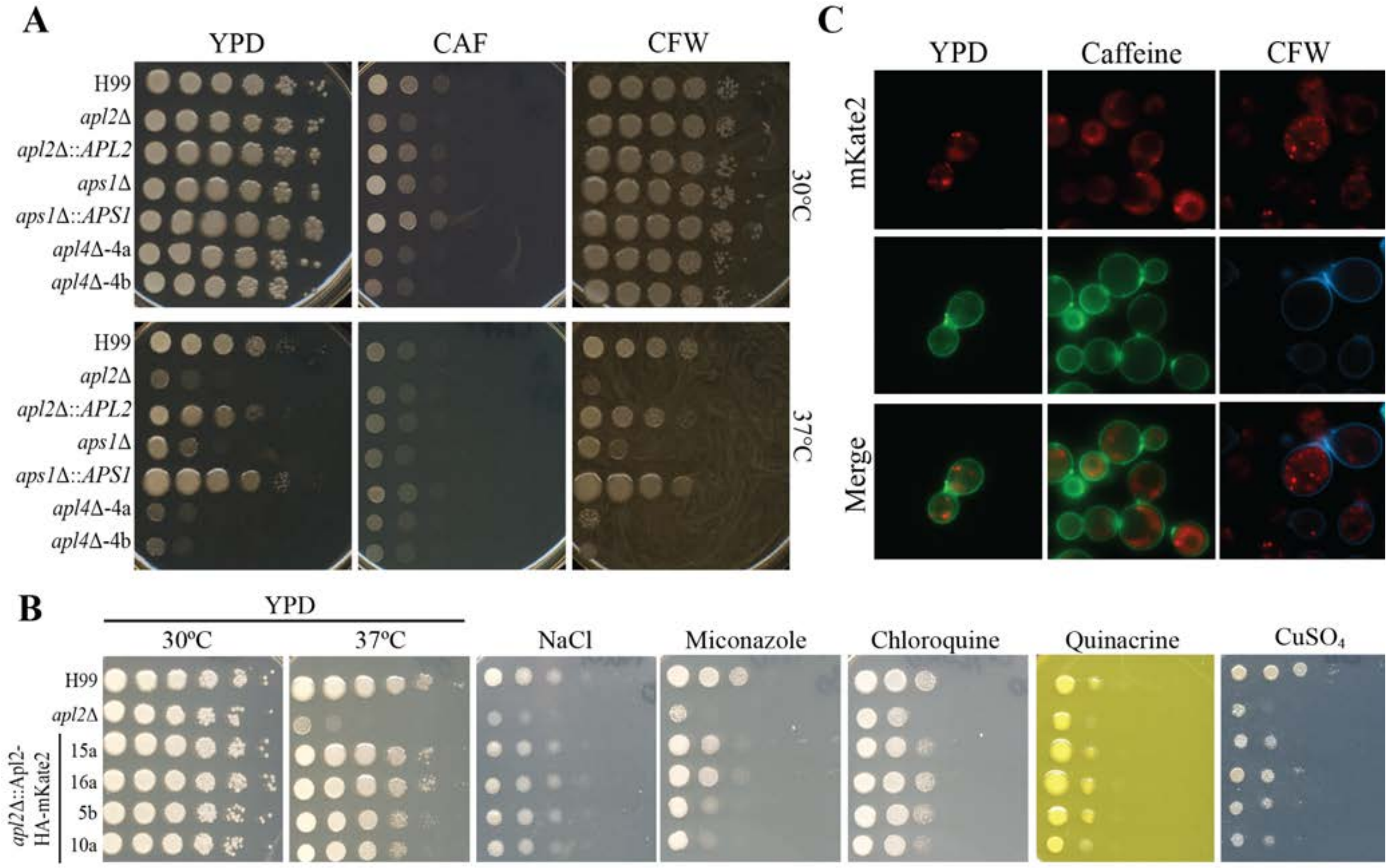

Fig S6. Cell wall stressors disrupt Apl2-HA-mKate2 localization and reduce growth. (A, B) Tenfold serial dilutions of the indicated strains were spotted onto solid YPD medium supplemented with or without caffeine (CAF, 1.5 mg/mL), calcofluor white (CFW, 3 mg/ml), sodium chloride (NaCl, 1. 5 mM), the azole antifungal miconazole (0.01 µg/mL), the vacuole function inhibitors chloroquine (6 mM) and quinacrine (1.6 mM), or copper sulfate ($CuSO_4$, 10 mM). All the plates were incubated at 30 °C or 37 °C for 2–4 days before being scanned. Representative images from at least three independent experiments are shown. (C) Wide-field fluorescence microscopy of the *apl2*Δ strains expressing the fusion protein Apl2-HA-mKate2:: *apl2*Δ. Cells were incubated in YPD with or without caffeine (1 mg/mL) or calcofluor white (CFW, 250 µg/ml) for 16-20 hours at 30 °C and stained with Solophenyl Flavine (SF) before imaging.

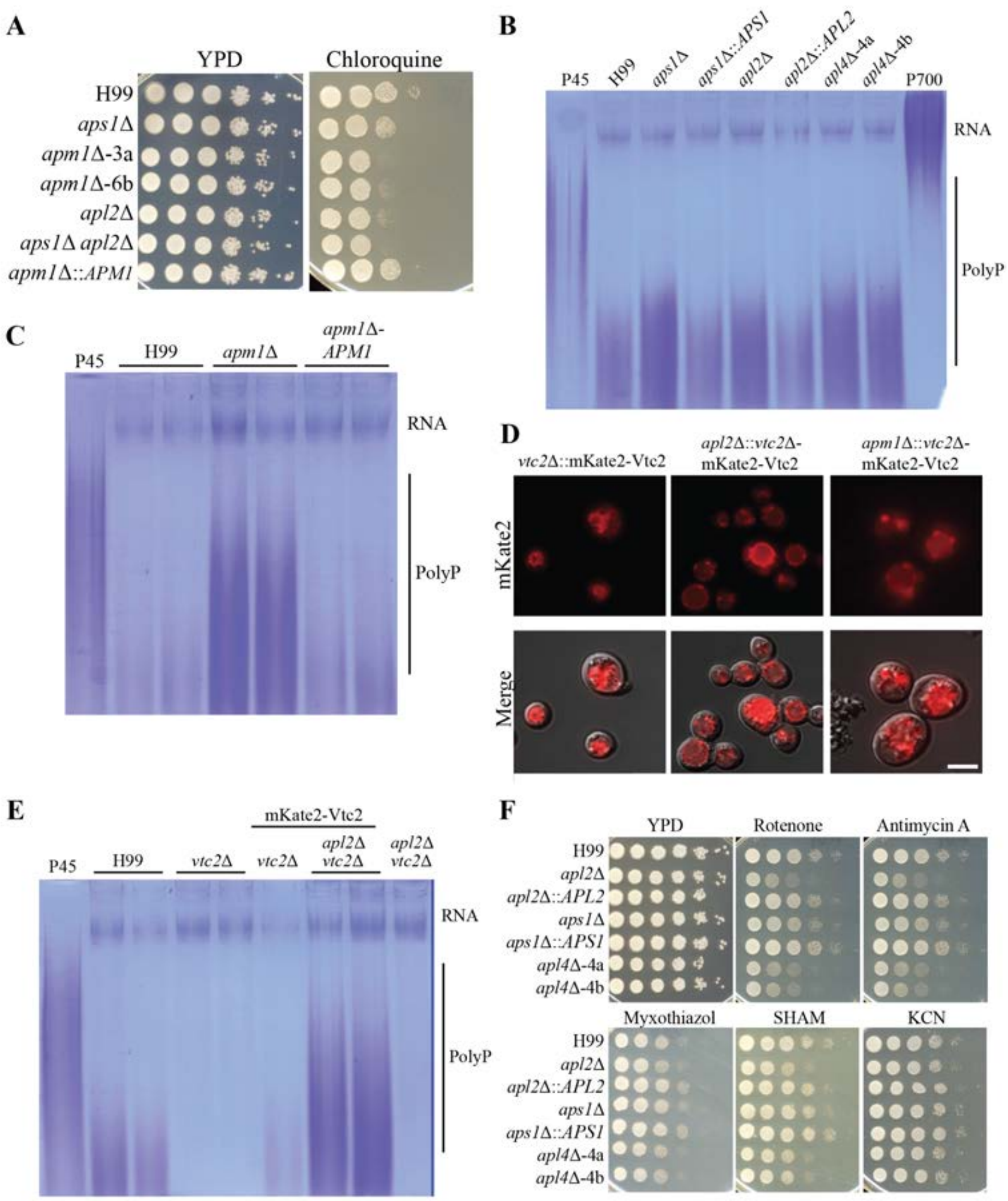

Fig S7. Defective AP-1 complex induces polyphosphate metabolism defects and abnormal subcellular distribution of the VTC complex subunit Vtc2. (A) Tenfold serial dilutions of the single deletion mutant strains (*aps1*Δ, *apm1*Δ-3, *apm1*Δ-6b, *apl2*Δ, *aps1*Δ *apl2*Δ) and the complement strain *apm1*Δ::*APM1*. Cells were spotted onto solid YPD medium supplemented with or without the vacuole function inhibitors chloroquine (6 mM). The plates were incubated at 30 °C for 2–4 days before being scanned. Representative images from at least three independent experiments are shown. (B, C) Detection of polyP on native acrylamide gels stained with toluidine blue O. Total RNA extracts (10 μg) from whole cell lysates of three biological replicates previously grown on YPD. Samples from the indicated strains were loaded on the gels using polyP types 45 and or 700 (P45 and P700, 10 μg) as standards. Densitometric analysis for regions containing polyP of the gel in S4B shown in Fig. 4D. The acrylamide gels are representative of two to three independent experiments. (D) Wide-field fluorescence microscopy of *vtc2*Δ strain and the AP-1 mutants (*apl2*Δ and *apm*1Δ) expressing mKate2-Vtc2. Cells were grown in CIM for 48 hours before imaging. Merged panels include differential interference contrast (DIC) images (not shown). Scale bars, 5 μm. (E) Detection of polyP on a native acrylamide gel stained with toluidine blue O as in (B and C). (F) Spot assays of tenfold serial dilutions of the indicated strains onto solid YPD medium with or without rotenone (25 μg/mL), antimycin A (5 μg/mL), myxothiazol (2.5 μM), salicylhydroxamic acid (SHAM, 5 mM), or potassium cyanide (KCN, 10 mM). The plates were incubated at 30°C for 2–3 days before being scanned. Images representative of at least three independent experiments.

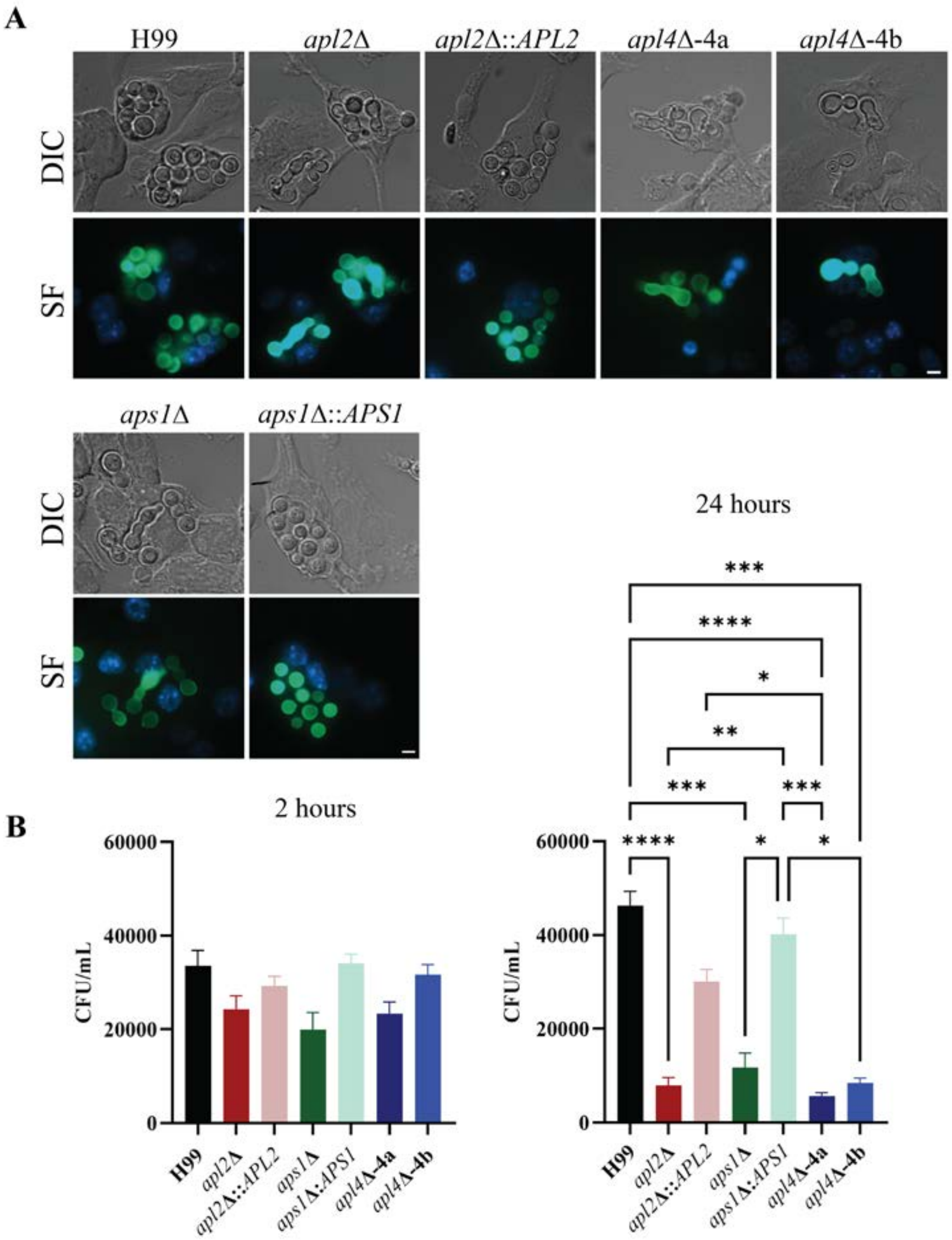

Fig S8. The AP-1 complex is required for intracellular survival of *C. neoformans* within macrophages *in vitro*. (A) Differential interference contrast (DIC) and wide-field fluorescence microscopy of phagocytosed wild-type (H99), single deletion mutants (*apl2*Δ, *aps1*Δ, *apl4*Δ-4a, *apl4*Δ-4b), and complemented strains (*apl2*Δ::*APL2*, *aps1*Δ::*APS1*). Opsonized *C. neoformans* cells with the anti-GXM monoclonal antibody 18B7 were incubated with J774A.1 macrophages at a multiplicity of infection (MOI) of 10:1. Fluorescence and DIC images of solophenyl flavine (SF) stained yeasts and macrophages fixed at 24 hours post-infection. Infections were carried out at 37 °C with 5% CO_2 for either 2 or 24 hours. Images are representative of at least three independent experiments, each observing more than 100 cells. Scale bars (10 µm). (B) Survival assays of *C. neoformans* strains following macrophage infection at 2 and 24 hours post-infection. The indicated strains were recovered from lysed macrophages, and colony-forming units (CFUs) were quantified on YPD plates after incubation at 30 °C for 2 days. Results are representative from three independent experiments ± SEM. Statistical significance shown was determined by ANOVA followed by Tukey *post hoc* test (*, P < 0.05; **, P> 0.01; ***, P> 0.001; ****, P < 0.0001). Corresponding data for the wild-type and single deletion mutants (*apl2*Δ, *aps1*Δ, *apl4*Δ-4a) are presented in Fig. 5C.

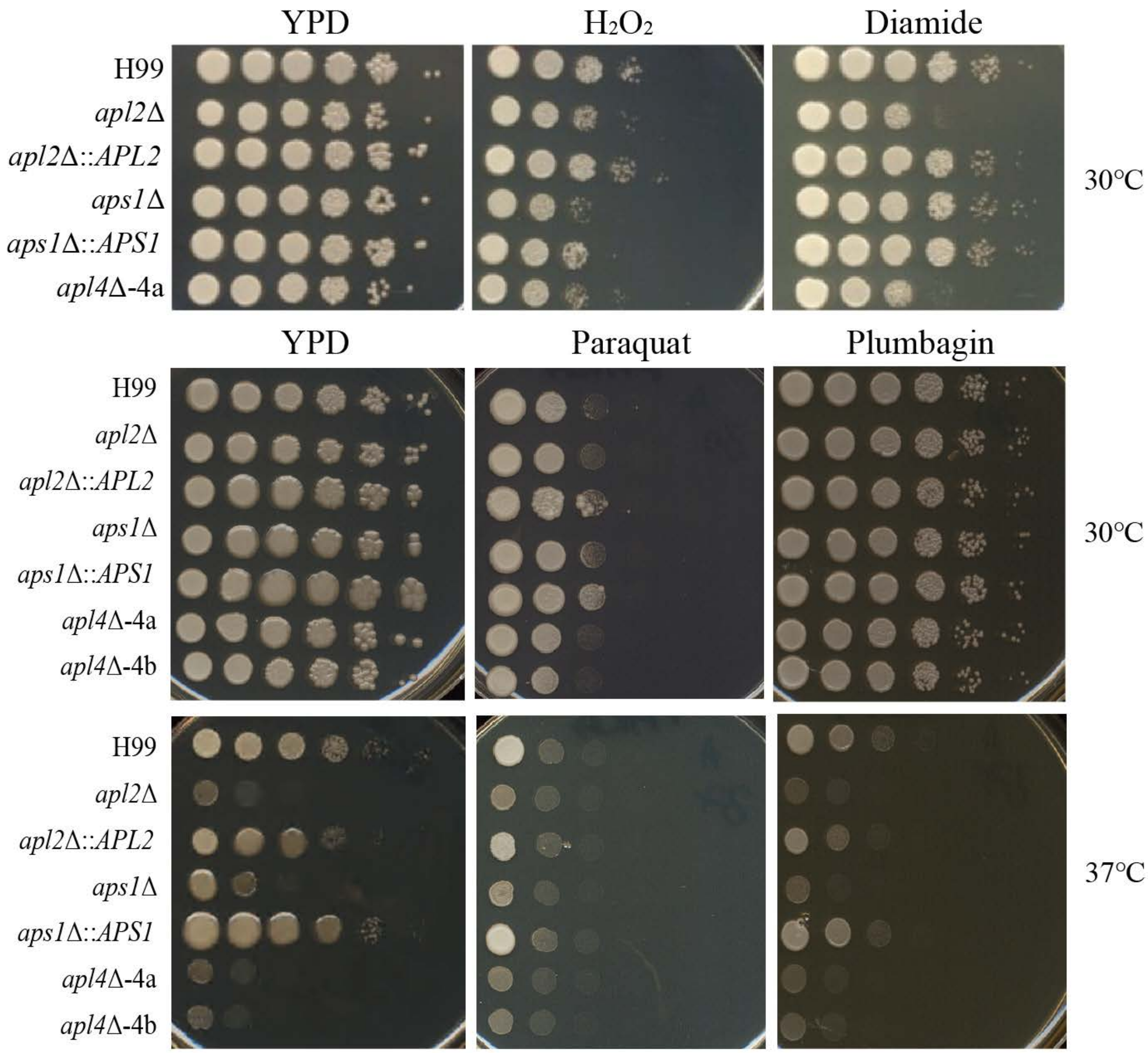

Fig S9. The AP-1 complex plays a minor role in reactive oxygen species (ROS) tolerance. Tenfold serial dilutions of the indicated strains were spotted onto solid YPD medium supplemented with or without the ROS stressors: oxygen peroxide (H_2O_2, 3mM), diamide (1 mM) or paraquat (0.7 mM) or plumbagin (100 μM). Plates were incubated at 30 °C or 37 °C for 2–4 days before being scanned. Representative images from at least three independent experiments are shown.

Supplemental Table S1. *C. neoformans* strains used in this study.

Strains		
**Name**	**Description, Genotype**	**Source, Reference**
Wild type	*C. neoformans* H99 wild-type strain, serotype A	J. R. Perfect
*aps1*Δ*-hm*	*aps1* deletion mutant in the KN99 background, *aps1*Δ*::NAT*	(Liu et al., 2008; McCluskey, 2011)
*apl2*Δ*-hm*	*apl2* deletion mutant in the KN99 background, *apl2*Δ*::NAT*	(Liu et al., 2008; McCluskey, 2011)
*apl4*Δ*-hm*	*apl4* deletion mutant in the KN99 background, *apl4*Δ*::NAT*	(Liu et al., 2008; McCluskey, 2011)
*apl2*Δ-2a, -2b	*apl2* deletion mutant in the H99 background, *apl2*Δ*::NAT*	This study
*apl4*Δ-4a, -4b	*apl4* deletion mutant in the H99 background, *apl4*Δ*::NAT*	This study
*aps1*Δ-7a, -11b	*aps1* deletion mutant in the H99 background, *aps1*Δ*::NAT*	This study
*apm1*Δ-3a, -6b	*apm1* deletion mutant in the H99 background, *apm1*Δ*::HYG*	This study
*aps1*Δ*::APS1-3a, -3b*	*aps1* deletion mutant strain expressing Aps1 at safe haven; *pEF1-Aps1::Hyg aps1*Δ*::NAT*	This study
*aps1*Δ*::APS1-4a, -4b*	*aps1* deletion mutant strain expressing Aps1 at safe haven; *pAPS1-Aps1::Hyg aps1*Δ*::NAT*	This study
*apl2*Δ*::APL2-1a, -3a*	*apl2* deletion mutant strain expressing Apl2 at safe haven; *pAPL2-Apl2::Hyg apl2*Δ*::NAT*	This study
*apl2*Δ*::APL2-HA*	*apl2* deletion mutant strain expressing Apl2 at safe haven; *pAPL2-Apl2-HA::Hyg apl2*Δ*::NAT*	This study
*apm1*Δ*::APM1-1a, -18b*	*Apm1* deletion mutant strain expressing Apm1 at safe haven; *pEF1-Apm1::NEO apm1*Δ*::HYG*	This study
*aps1*Δ *apl2*Δ*-3,-5*	*aps1* and *apl2* double deletion mutant in the H99 background, *aps1*Δ*::NAT apl2*Δ*::HYG*	This study

Sec61-GFP mCherry-Cas35-8a	PGal7::PKA1 strain expressing SEC61-GFP and mCherry-Cas35; *mCherry-CAS35::HYG*	This study
mCherry-Cas35-3a, -4a	H99 wild-type strain with mCherry-Cas35 expressed from its endogenous locus; *mCherry-CAS35::NEO*	This study
mCherry-Cas35::*apl2*Δ-4a, 1b	*apl2* deletion mutant strain expressing mCherry-Cas35; *mCherry-CAS35::NEO, apl2*Δ*::NAT*	This study
mCherry-Cas35::*aps1*Δ-2, -3	*aps1* deletion mutant strain expressing mCherry-Cas35; *mCherry-CAS35::NEO, aps1*Δ*::NAT*	This study
Aph1-DsRED	H99 wild-type strain with Aph1-DsRED expressed from its endogenous locus; *APH1-DSRED::NEO*	(Hu et al., 2017)
Aph1-DsRED::a*pl2*Δ-1a, 7b	*apl2* deletion mutant strain expressing Aph1-dsRED; *APH1-DsRED::NEO, apl2*Δ*::NAT*	This study
*vtc2*Δ *-1a*	*vtc2* deletion mutant in the H99 background, *vtc2*Δ*::NAT*	(Sánchez-León et al., 2025)
*apl2*Δ *vtc2*Δ*-19, -20*	*apl2* and *vtc2* double deletion mutant in the H99 background, *vtc2*Δ*::NAT apl2*Δ*::HYG*	This study
mKATE2-Vtc2::*vtc2*Δ	*vtc2* deletion mutant strain expressing mKATE2-Vtc2 targeted at safe haven; *pEF1-mKATE2-VTC2::NEO vtc2*Δ*::NAT*	(Sánchez-León et al., 2025)
mKATE2-Vtc2::*vtc2*Δ *apl2*Δ-8, -9	*vtc2 apl2* double deletion mutant strain expressing mKATE2-Vtc2 targeted at safe haven; *pEF1-mKATE2-VTC2::NEO vtc2*Δ*::NAT apl2::HYG*	This study
Wild type (sc)	*S. cerevisiae* SC288 wild-type strain	
*aps1*Δ-sc	*aps1* deletion mutant in *S. cerevisiae* BY4741*, aps1*Δ*::KANMX*	Yeast Knockout Collection

*apl2*Δ-sc	*apl2* deletion mutant in *S. cerevisiae* BY4741, *apl2*Δ*::KANMX*	Yeast Knockout Collection

Supplemental Table S2. Plasmids and primers used for PCR amplification of the gene deletion constructs and for mutant screenings.

Primer Name	Sequence 5'→3'	Reference
ES161	Tttgaagttttctgtggagatc	This study
ES192	ACAATCGGCTGCTCTGATG	This study
ES193	AATATCACGGGTAGCCAACG	This study
ES408	ACCGTAATTCGGCCAAGGGTTC	This study
ES409	TCTTCGGAGCAAAAGAGCTTGC	This study
ES412	CTACACGATCCCCTCCTCAA	This study
ES415	AAGGGTACGCCTCCAGGTAT	This study
ES423	ACTTGCTAGGCGAACAAGGA	This study
ES424	GCCGAAATATCGATCCAAGA	This study
ES425	CAAACATTGTCAGGCCCTTT	This study
ES426	TCCTTGTGATCGACTTGCTG	This study
ES446	ATGCTCAACTATGTCATGCTCG	This study
ES466	TCTTCTCACAGGGAGTCTGACA	This study
ES467	AACGCTAAAATTGGGCCATA	This study
ES468	GAGAACCCGTAAGCATCTGTTC	This study
ES469	CCTTTCAAAAAGGGGGCTTTCC	This study
ES470	TGCTCCAGGTCATCTGTTTG	This study
ES471	ATCTACTTGAATACCTGGTATT	This study
ES472	GCGCCTATGAAAGCTTGTTC	This study
ES473	TGTCCAAACTGACCAACCAA	This study
ES476	TAATACGACTCACTATGTTCTTTGGCATTTGAGTGC	This study

ES477	GCTTCGAGacaACAACTTGCCTAGTGGTTTCCATGTAATG	This study
ES478	GTTGTtgtCTCGAAGCATG	This study
ES479	ATAGTGAGTCGTATTACGCG	This study
ES480	Gagctcagagaccatgtgtttgaagttttctgtggagatc	This study
ES481	TTGAGCGTGCTTCATTGG	This study
ES482	TTGAGTCCATGGCTGCGA	This study
ES483	TAATACGACTCACTATAATATTAGAGGGTAGAAAGAACAAGC	This study
ES484	GCTTCGAGacaACAACTATCGGTCTATGTCGTCTATAGTGC	This study
ES487	ACTTCGCCAACAGCTGAACT	This study
ES488	CGCTTTGACGAAAGTGTTGA	This study
ES491	ACGGATGACTGTTTGGGAAG	This study
ES492	GCAACATAGGGGTGCTTTGT	This study
ES493	GGACAGATAGACACGTGTAACC	This study
ES494	aaccggccaatgaagcacgctcaaCTTACCAGCTGTCCGTTTTCC	This study
ES495	cacatcctcgcagcCATGGACTCAAGCATGACAGCATGACATTCC	This study
ES496	TCGCGAGTTCCTTATTATCTGG	This study
ES498	CGGATCTGATTCAACCCTGTCAAC	This study
ES570	Gcccctgaccaaggtgtt	This study
ES571	Gcgttaacgttggtgacctc	This study
ES573	CCTTACGATGTCCCTGATTACGCTggatcccacatggtctctgagctcat	This study
ES581	cacagaaaacttcaaaATGTTGTATCTCACAGCAGACA	This study
ES583	CAGGGACATCGTAAGGGTATGCTTTTCTGCGCGCAGA	This study
ES630	TCCATCCAGGGTCGTACCTA	This study
ES631	gcagcCATGGACTCAAGATTAGGGCTGATGCTTGAGAG	This study
ES632	aatgaagcacgctcaaTTTGTTAGAAATCGATGTGTCT	This study
ES633	TTTGATGTCCCAGGTCATTG	This study
ES634	GATGCCTCCGCTCGAAGTAG	This study
ES635	GATGTAGGAGGGCGTGGATA	This study
ES644	aatgaagcacgctcaaATGAATAACTGAAAATGTATGTT	This study
ES645	gcagcCATGGACTCAATTTGAACGCCTTTTTTCCGGGA	This study

ES646	GCCGGAGAAGGATTGGTGAA	This study
ES647	gcagcCATGGACTCAACTGCAGGTATGGTCAGCTC	This study
ES648	aatgaagcacgctcaaGACAGTCCAAAGTTCAGAG	This study
ES649	GCCACCCAGGTATCTTGCTT	This study
ES650	GACAAGACTGAGGCCATGCT	This study
ES651	Atgactcaccttgggcagtg	This study
ES667	cacagaaaacttcaaaATGGCCAGTCTCGGTACG	This study
ES668	GCTTCGAGacaACAACCATCCACCCAGATGACCCA	This study
ES672	GATGTCCCTGATTACGCTTAGGtaccATATGGGTTTCCGGCGTCT	This study
ES673	CGTAATCAGGGACATCGTAAGGGTAAAACAGTCCCAACAAATCATCTTGAGC	This study
UQ2962	GGGTATGCCACAGATGCAGAT	(Arras et al., 2015)
UQ2963	TTGGATCCTCAATTGTCTCCT	(Arras et al., 2015)
UQ1768	TCAGCAACGCCGTTGAATCCT	(Arras et al., 2015)
UQ3348	ACTGGTGAGTACTCAACCAAG	(Arras et al., 2015)
**Plasmids**		
Name	Description	Reference
pESL018-2	A plasmid vector encoding the *C. neoformans* heme sensor (CnHS), comprising a bimolecular mKATE-2::eGFP fluorescent protein tag, harbouring the HYG (hygromycin) resistance cassette	(Bairwa et al., 2019)
pESL018-2-pAPS1-Aps1	Complementation vector with the *APS1 gene* sequence of *C. neoformans* H99 containing the safe haven region*; pEF1-APS1::HYG*	This study
pESL018-2-pEF1-Aps1	Complementation vector with the *APS1 gene* sequence of *C. neoformans* H99 containing the safe haven region*; pEF1-APS1::HYG*	This study
pESL018-2-pAPL2-Apl2	Complementation vector with the *APL2 gene* sequence of *C. neoformans* H99 containing the safe haven region*; pAPL2-APL2::HYG*	This study

pESL018-2-pAPL2-Apl2-HA	Complementation vector with the *APL2 gene* sequence of *C. neoformans* H99 containing the safe haven region*; pAPL2-APL2-HA::HYG*	This study
pESL018-9	A plasmid vector encoding the *C. neoformans* optimized fluorescent protein tag mKATE-2; *pEF1-mKATE2* harbouring the NEO (Neomycin) resistance cassette	This study
pESL018-9-pEF1-Apm1	Complementation vector with the *APM1 gene* sequence of *C. neoformans* H99 containing the safe haven region*; pEF1-APM1::NEO*	This study
pESL018-9-HA-mKATE2	Vector containing mKATE2 with N-terminal HA tag*; pEF1-HA-mKATE2::NEO*	This study
pESL018-9-Cps1-HA-mKATE2	Vector with the *CPS1 gene* sequence of *C. neoformans* H99 c-terminally linked to HA-mKATE2*; pEF1-CPS1-HA-mKATE2::NEO*	This study
